# Non-antibiotic therapies for multidrug-resistant gastrointestinal infections: an overview of the use of probiotics, natural compounds, and bacteriophages

**DOI:** 10.3389/frabi.2025.1554061

**Published:** 2025-05-06

**Authors:** Manuela Oliveira, Áurea Madureira-Carvalho, Ricardo Jorge Dinis-Oliveira, Diana Dias da Silva

**Affiliations:** ^1^ Associate Laboratory i4HB - Institute for Health and Bioeconomy, University Institute of Health Sciences - Cooperativa de Ensino Superior Politécnico e Universitário (CESPU), Gandra, Portugal; ^2^ Applied Molecular Biosciences Unit (UCIBIO), Forensics and Biomedical Sciences Research Laboratory, University Institute of Health Sciences, One Health Toxicology Research Unit (1H-TOXRUN), Instituto Universitário de Ciências da Saúde da Cooperativa de Ensino Superior Politécnico e Universitário (IUCSCESPU), Gandra, Portugal; ^3^ Research Unit on Applied Molecular Biosciences (UCIBIO), Translational Toxicology Research Laboratory, University Institute of Health Sciences (1H-TOXRUN, IUCS-CESPU), Gandra, Portugal; ^4^ Department of Public Health and Forensic Sciences and Medical Education, Faculty of Medicine, University of Porto, Porto, Portugal; ^5^ FOREN - Forensic Science Experts, Lisbon, Portugal; ^6^ Network of Chemistry and Technology/Laboratory for Green Chemistry (REQUIMTE/LAQV), Escola Superior de Saúde (ESS), Polytechnic of Porto, Porto, Portugal; ^7^ Associate Laboratory 4HB - Institute for Health and Bioeconomy, University of Porto, Porto, Portugal; ^8^ Applied Molecular Biosciences Unit (UCIBIO), Laboratory of Toxicology, Faculty of Pharmacy, University of Porto, Porto, Portugal

**Keywords:** alternative therapies, antibiotic resistance, biofilm disruption, gut microbiota, immune modulation, microbial interactions, natural compounds

## Abstract

The worldwide increasing frequency and severity of multidrug-resistant gastrointestinal (MDR-GI) infections not only raises awareness of the debilities of conventional antibiotic treatments but also highlights the demand for alternative interventions. One of these alternatives is probiotics, harmless bacteria that compete with pathogenic species, which have been considered beneficial due to their therapeutic potential since they strengthen the mucosal barrier and modulate the host immune response. Other natural compounds (e.g., polyphenols, flavonoids, and essential oils) present diverse antimicrobial mechanisms, which are promising alternatives to mitigate resistant pathogens. Finally, bacteriophages, viruses that target specific bacteria, constitute a precise approach in which MDR bacteria are lysed or disrupted by the biofilms formed during colonization without compromising the normal gut microbiome. Therefore, the present manuscript provides an integrated perspective on alternative non-antibiotic therapies to manage MDR-GI infections; for this purpose, it covers aspects such as their action mechanisms, current clinical applications, and the challenges that limit their broader application in clinical practice. The potential of combining these approaches or personalizing infection treatments adjusted to patients’ microbiome profiles is also discussed, aiming to enhance efficacy and reduce resistance risks. Finally, the importance of continued research and development to optimize these alternatives is also debated, addressing aspects such as the need to surpass regulatory barriers and conducting large-scale clinical trials to establish the safety and efficacy of these non-antibiotic alternatives. This overview of the current knowledge contributes to the ongoing efforts to develop sustainable strategies to combat MDR-GI infections and reduce the global burden of antibiotic resistance.

## 1 Introduction

The ever-growing frequency and severity of MDR-GI infections constitute a pressing global health concern, mainly in low- and medium-income countries (LMICs) (Browne et al., 2020). Over the past decades, several foodborne bacteria have become important etiological agents of GI infections (e.g., *Clostridioides difficile*, *Helicobacter pylori*, *Listeria monocytogenes*, certain species of *Campylobacter* spp., *Salmonella* spp., and *Shigella* spp.) frequently associated with resistance to multiple classes of antibiotics (**Supplementary Table 1**) (Grudlewska-Buda et al., 2023). Each of these bacterial species present a particular mechanism of antibiotic resistance, but all pose diverse adverse clinical implications, constraining treatment outcomes (Hasanuzzaman et al, 2024). Resistance emergence and dissemination are often driven by factors such as the overuse and misuse of antibiotics both in healthcare and agriculture, the persistence of antibiotics in ecosystems, the rapid mutation and evolution of bacterial populations to adjust to ever-changing therapeutical approaches, and inadequate global infection control measures (Oliveira et al., 2024). Moreover, the MDR-GI problem infection is further exacerbated by the high transmissibility of these resistant pathogens, which threaten individual health and undermines public health (Salam et al., 2023). The most common consequences of MDR-GI infections often include prolonged illness, higher healthcare costs, and elevated mortality rates, particularly in vulnerable populations, further contributing to economic impairment in already debilitated regions (SChinas et al., 2023).

Since its development in the 1940s, antibiotics have been considered the cornerstone to combat bacterial infections (Cook and Wright, 2022). Nevertheless, the effectiveness of these drugs against MDR-GI bacteria and other pathogens has increasingly been compromised by the rise of these resistance patterns (Vanegas-Múnera and Jiménez-Quiceno, 2020). Moreover, antibiotic therapy often disrupts the normal gut microbiota, reducing species diversity and disrupting regular metabolic and immunological functions, leading to aggravated infections and conditions such as antibiotic-associated diarrhea or recurrent *C. difficile* infections (Dahiya and Nigam, 2023). Also, the exacerbated reliance on broad-spectrum antibiotics further promotes resistance emergence and, consequently, limits treatment options for future infections (Hotinger et al., 2021). Finally, the pharmaceutical industry’s stagnation in developing new antibiotic classes further aggravates this problem, leaving clinicians with limited practical possibilities to manage these complex bacterial infections (Cook and Wright, 2022). Altogether, the above-presented limitations highlight the urgent need for innovative and effective therapeutical alternatives that can address the immediate problem of MDR-GI infections and the long-term effects of antimicrobial resistance (AMR).

The research and development of alternative therapies will overcome the limitations posed by the current antibiotic regimens. Over time, non-antibiotic strategies have gained significant attention since they offer action mechanisms distinct from conventional antibiotics and may change the therapeutical approach to MDR-GI infections (Murugaiyan et al., 2022). Among the most common alternatives are probiotics, natural compounds, and bacteriophages (Murugaiyan et al., 2022). All these formulations are aligned with the goals of antimicrobial stewardship programs, which advocate the reduction of antibiotic dependency and the mitigation of resistance emergence and dissemination (Majumder et al., 2020). Additionally, these new alternatives are prone to be tailored to specific pathogens and patient needs, contributing to the growing field of personalized medicine (Konwar et al., 2022).

While the primary focus of the present review is on predominant pathogens such as *Salmonella*, *E. coli*, and *Campylobacter*, it is noteworthy to mention that less common opportunistic infections can pose significant health challenges. Infections caused by *Staphylococcus aureus*, *Pseudomonas aeruginosa*, and *Enterococcus faecalis* represent a smaller fraction in gastrointestinal cases (Elashiry et al., 2023; Gupta and Dey, 2023; Liu et al., 2024) must be pointed out due to their potential to exploit compromised immune systems or disrupted gut microbiomes. These pathogens underline the diverse bacterial landscape that affects gastrointestinal health and warrant attention for comprehensive disease management and prevention strategies (Dey, 2024).

Moreover, vaccines against enteric pathogens like *Shigella*, Enterotoxigenic *E.* coli (ETEC), and *Salmonella* are essential to prevent many diarrheal diseases, especially in LMICs (Seo et al., 2020; Khalil et al., 2021). While progress has been made, several challenges remain in developing broadly protective vaccines due to strain heterogeneity and limited efficacy in endemic regions (Seo et al., 2020). Novel approaches, such as the Multiepitope fusion antigen - MEFA platform and combined vaccines like ShigETEC, show promise in addressing these issues (Seo et al., 2020; Harutyunyan et al., 2020). Vaccination could be a cost-effective and equitable means of primary prevention, complementing other interventions such as improved sanitation (Khalil et al., 2021). A multi-pathogen vaccine targeting Shigella, ETEC, and Campylobacter could address about one-third of diarrhea cases in children and align with WHO’s preferred product characteristics (Walker et al., 2021). Continuous research and development efforts will allow to overcome challenges and accelerate the availability of effective vaccines for these enteric pathogens.

Through a comprehensive literature revision, the present review provides an overview of the available non-antibiotic alternative therapies for MDR-GI infections - probiotics, natural compounds, and bacteriophages. In each case, aspects such as their potential, limitations, and future directions for research and development were addressed. Bearing these objectives in mind, the discussion begins with the sections “Probiotics”, “Natural compounds”, and “Bacteriophages”, which cover their action mechanisms, clinical applications and challenges associated with their use. Afterward, the manuscript explores integrative approaches, such as the synergistic effects of combining these alternatives, as well as the recent developments, such as personalized medicine, based on the study of individual microbiome profiles. Finally, future directions are outlined, emphasizing advancements in understanding microbiome-pathogen interactions, developing novel delivery systems, and the need for large-scale clinical trials and regulatory frameworks.

## 2 Material and methods

The selection of manuscripts for this review followed a structured and systematic approach to ensure methodological transparency and rigor. Initially, a total of 2,543,872 manuscripts were retrieved from public article databases, including PubMed (U.S. National Library of Medicine) and Google Scholar, using a comprehensive set of keywords such as “Non-Antibiotic Therapies”, “Multidrug-Resistant Pathogens”, “Gastrointestinal Infections”, “Probiotics”, “Natural Compounds”, “Bacteriophages”, “Limitations”, and “Future Directions”. After the removal of duplicate entries, 2,543,336 unique records remained.

The first screening stage involved an initial evaluation based on the relevance of the title's and abstract's relevance. Studies not directly related to non-antibiotic therapies for MDR-GI infections were excluded from further analysis, significantly reducing the number of manuscripts considered for full-text assessment.

For the next stage, 536 studies underwent a thorough full-text review based on specific inclusion criteria. Only studies published between 2019 and 2024 were considered to ensure that the most recent research findings were incorporated. Manuscripts selected for inclusion had to be peer-reviewed and published in high-impact journals classified within the Q1 and Q2 categories. Studies with robust methodologies, high-quality data, and practical relevance were prioritized, and full-text availability was required to evaluate the study findings comprehensively.

During the eligibility assessment, several exclusion criteria were applied. Manuscripts published before 2019 were removed to avoid outdated research. Studies lacking peer review (e.g., preprints and conference abstracts) were also excluded to maintain scientific rigor. Further, manuscripts with methodological flaws, insufficient statistical validation, or weak experimental design were not considered. Additionally, those without full-text availability were omitted from the analysis.

**Figure 1:**
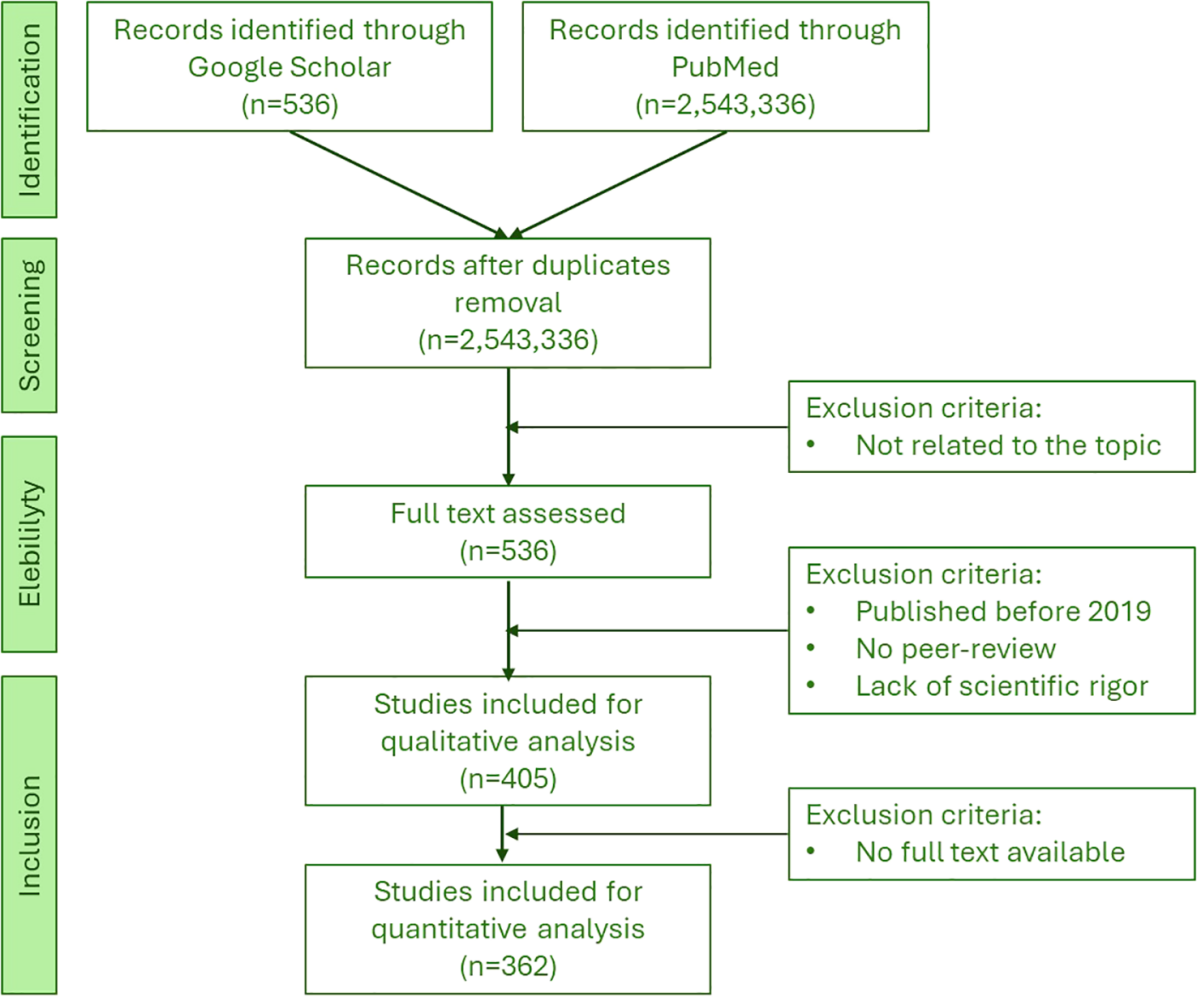
Schematic representation of the flowchart of the searched articles and the inclusion and exclusion criteria.

Following this rigorous selection process, 405 manuscripts were included for qualitative synthesis, while 362 studies met all criteria for quantitative analysis. The comprehensive selection method in the present manuscript ensures that only the most relevant, high-quality, and scientifically rigorous studies were incorporated, providing an updated and reliable review of non-antibiotic therapies for multidrug-resistant gastrointestinal infections (**Figure 1**).

## 3 Probiotics

Probiotics, defined as live microorganisms that confer health benefits when adequately administered, can be used for GI infections and other conditions (Silva et al., 2020). These non-antibiotic alternatives offer the dual advantage of mitigating multidrug-resistant (MDR) pathogens and promoting the integrity and functionality of the gut microbiome (Jain et al., 2023). Probiotics’ action mechanisms are diverse and include enhancing mucosal barrier function, competitive inhibition of pathogens, and modulation of the immune system (Van Zyl et al., 2020; Mazziotta et al., 2023). Probiotics prevent and ameliorate digestive disorders, allergies, and inflammatory conditions (Plaza-Diaz et al., 2019). Since they interfere with the gut-brain axis (Dahiya and Nigam, 2023), the gut-liver axis, and the gut-lung axis (Stavropoulou and Bezirtzoglou, 2020), they potentially influence systemic health. The probiotics’ attachment to intestinal epithelium influences their functionality, with factors such as sortase A (i.e., a transpeptidase enzyme responsible for anchoring surface proteins to the bacterial cell wall pathogen’s being involved in bacterial virulence, adherence, and immune evasion), playing a significant role in this process (Javanshir et al., 2021). Probiotics also produce antimicrobial compounds, regulate fecal enzymatic activities, and generate short-chain fatty acids that affect both intestinal and peripheral tissues (Plaza-Diaz et al., 2019).

**Figure 2:**
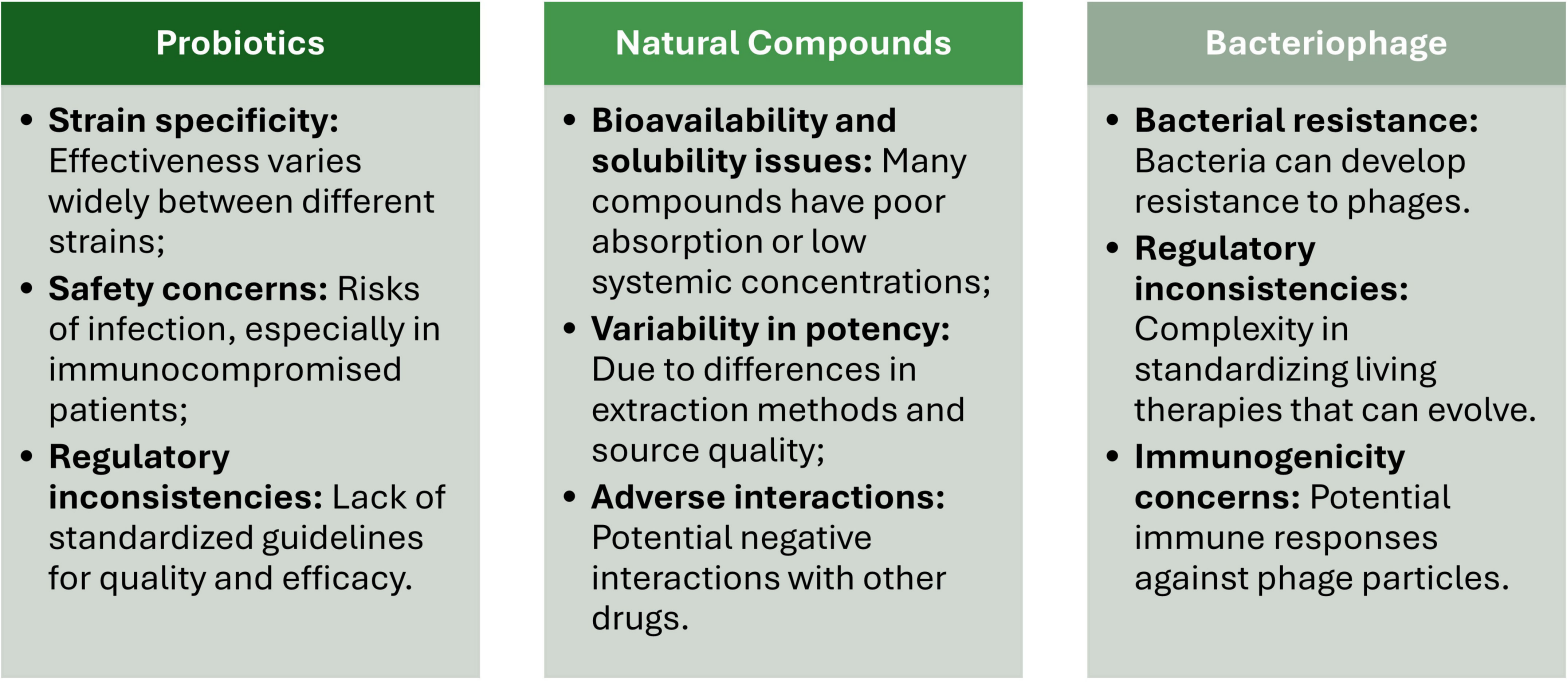
Main actions of probiotics, natural compounds, and bacteriophages in managing multidrug-resistant gastrointestinal infections.

One key mechanism of probiotics’ action is the competitive inhibition of pathogenic bacteria. Probiotic bacteria and yeast (e.g., *Lactobacillus* spp., *Bifidobacterium* spp., *Streptococcus thermophilus*, *Escherichia coli* Nissle 1917, *Enterococcus* spp., *Saccharomyces boulardii*) occupy attachment sites on gut epithelial cells, preventing pathogens from adhering to and colonizing these surfaces (Plaza-Diaz et al., 2019; Javanshir et al., 2021). Moreover, probiotics also compete for essential nutrients, limiting the resources available to those harmful microbes (Tang and Lu, 2019) and produce antimicrobial substances (e.g., organic acids, bacteriocins, and hydrogen peroxide) that lower local pH levels or disrupt the cell membranes of pathogenic bacteria, impeding their growth and activity (Khaneghah et al., 2020) (**Figure 2**).

Enhancing the mucosal barrier is another important function of probiotics. The mucosal epithelial cells are the first defense against microbial invasion (Camilleri, 2021). Probiotics are known to strengthen this barrier by stimulating the production of mucins, which protect epithelial cells from direct microbial contact (Gou et al., 2022). Also, they improve the integrity of tight junctions between epithelial cells, sealing the space between adjacent epithelial cells and making it more difficult for bacteria to invade and disrupt host tissues (Rose et al., 2021). Combined, these two aspects reduce intestinal permeability and prevent the translocation of pathogens and their toxins into the systemic circulation, containing the infection locally. This process is achieved by activating macrophages and dendritic cells, stimulating immunoglobulin A (IgA) production, and regulating cytokines release (Maldonado et al., 2019; Mazziotta et al., 2023) (**Figure 2**). Moreover, probiotics are also responsible for the delicate balance between pro- and anti-inflammatory signals required to resolve infections without exacerbating GI inflammation (Colletti et al., 2023) (**Figure 2**).

The therapeutic potential of probiotics is particularly evident in infections caused by *C. difficile* and *H. pylori* (Keikha and Karbalaei, 2021; Pal et al, 2023). In the case of *C. difficile*, recurrent infections constitute a significant challenge in clinical management, mainly due to the disruption of gut microbiota by antibiotics. Recent studies have demonstrated that some probiotic strains, such as *Lactobacillus* spp. and *Saccharomyces boulardii*, can restore microbiota balance, reduce toxin production, and decrease recurrent infection incidence. These effects are enhanced when probiotics are used as a co-adjuvant to antibiotic therapy (Pal et al., 2023), by potentially interfering with quorum sensing systems, reducing virulence, and disrupting toxin production, motility, and adherence. These aspects demonstrate the potential use of these alternatives to synergize with conventional treatments (Gunaratnam et al., 2021). In the case of *H. pylori* infections, probiotics, such as *Lactobacillus reuteri*, *Lactobacillus rhamnosus* and *Bifidobacterium bifidum*, have demonstrated the ability to inhibit *H. pylori* adhesion to gastric epithelial cells and reduce the infection-associated inflammation (Keikha and Karbalaei, 2021). Moreover, probiotics alleviate side effects commonly associated with the antibiotics used to treat *H. pylori*, such as diarrhea and gut dysbiosis, improving patient compliance with treatment regimens (Liang et al., 2022; Fiorani et al., 2023).

The commercial probiotic market has expanded rapidly, with various products available in shops and pharmacies (Liang et al., 2022). However, there are potential limitations and safety concerns associated with these products, including pathogenic contamination and inaccurate labeling (Liang et al., 2024). Developing high-quality probiotic formulations requires careful strain selection, safety assessments, and efficacy studies (Grumet et al., 2020). The introduction of DNA sequencing technologies has contributed to a deeper understanding of gut microbiota composition and its role in human health (Ballini et al., 2023). While the probiotic market continues to grow, with products ranging from yogurt to nutritional supplements, the papers reviewed do not mention any significant probiotic products based on rigorous studies entering the commercial market in the last five years.

**Figure 3:**
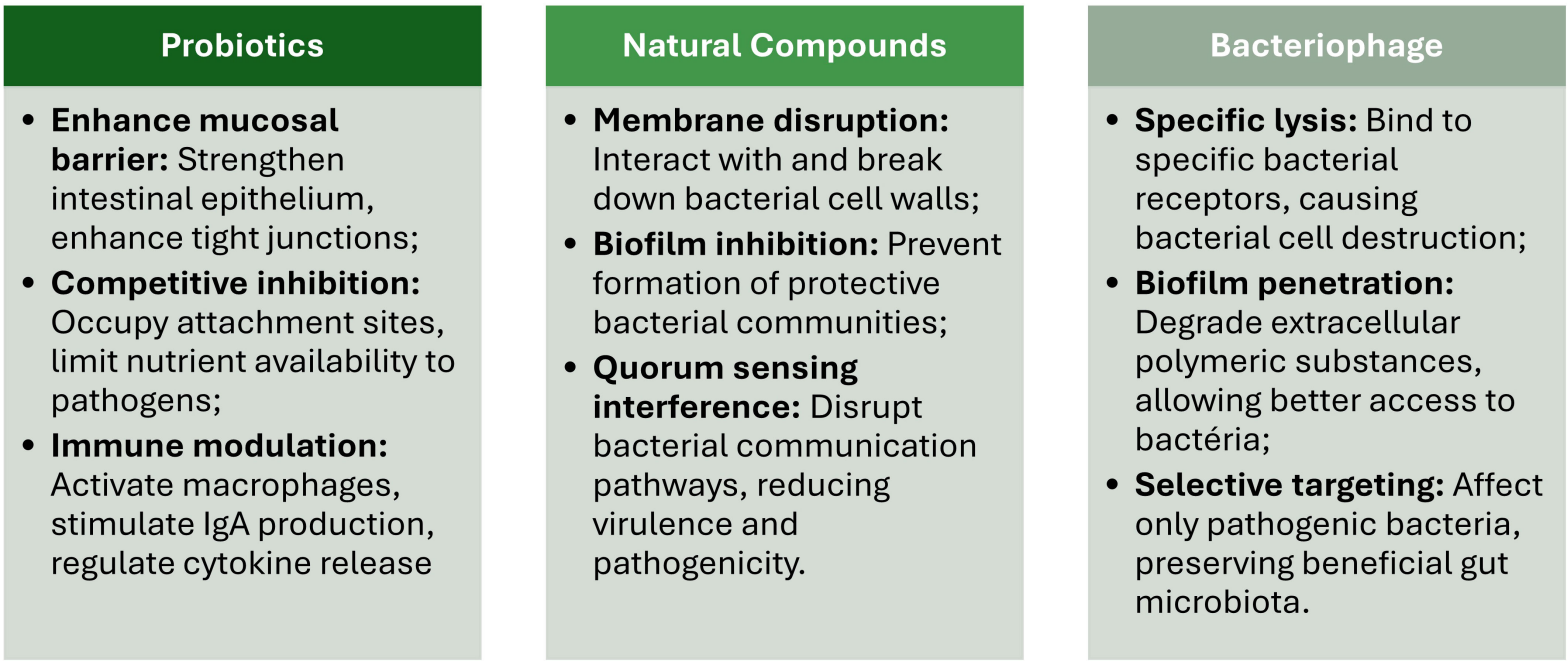
Main potential risks, side effects, and challenges in the clinical translation of probiotics, natural compounds, and bacteriophages.

While probiotics show potential in MDR-GI infection management, their clinical translation faces several challenges and risks. While certain Clostridium species may have probiotic effects, concerns exist regarding their safety (Guo et al., 2020). The expanding range of probiotic candidates necessitates improved quality assurance techniques to ensure dose, viability, and functional integrity (Cunningham et al., 2021). Safety considerations include potential adverse events, especially in vulnerable populations, emphasizing the need for whole genome sequencing to identify virulence, toxin, and antibiotic resistance genes (Merenstein et al., 2023). Therefore, a careful evaluation of risk-benefit ratios and patient selection should be performed during clinical translation, with personalized microbiome therapy potentially offering a path to successful treatment (Fong et al., 2020) (**Figure 3**).

Several challenges must be addressed before fully integrating probiotics into clinical practice (Stavropoulou and Bezirtzoglou, 2020). These challenges include knowledge gaps in defining optimal regimens for specific patient subgroups, regulatory issues concerning product safety and quality, and surpassing challenges of cross-colonization (Pell et al., 2019). Probiotics’ efficacy depends on strain, dose, host immune system, and therapy duration (Biswasroy et al., 2021). To address stability issues in different gastrointestinal tract compartments and improve targeted delivery, novel encapsulation techniques, such as stimuli-responsive polymers, are being developed (Garcia-Brand et al., 2022). While probiotics have shown potential in preventing conditions such as necrotizing enterocolitis in premature infants (Underwood, 2019), more extensive clinical studies are required to establish strong evidence for their efficacy and safety across various applications (Pell et al., 2019; Stavropoulou and Bezirtzoglou, 2020) (**Figure 3**).

Regulatory issues (e.g., probiotics are regulated differently across countries and regions, leading to inconsistencies in their classification, approval, and monitoring) and standardization issues (e.g., inconsistent quality control, dosing standards, and efficacy evaluation) further complicate the adoption of probiotics into the clinical practice (Silva et al., 2020; Britton et al., 2021) (**Figure 3**).

## 4 Natural compounds

Natural compounds are bioactive substances produced by plants to defend themselves against diverse threats (e.g., microorganisms, insects, and environmental stresses) (George and Brandl, 2021). They are frequently found in leaves, fruits, and other plant organs and exhibit antimicrobial effects against a wide range of pathogens, such as those associated with MDR-GI infections (Khameneh et al., 2021). Among the most well-studied natural compounds are polyphenols, flavonoids and essential oils, each presenting unique antimicrobial activity action mechanisms (**Supplementary Table 2**).

Polyphenols, such as tannins and catechins, have been reported as potent antimicrobial agents that disrupt bacterial membranes, bind to proteins, and interfere with several key metabolic pathways (Elkhalifa et al., 2024). For instance, while curcumin exhibits anti-inflammatory and antimicrobial properties by inhibiting bacterial adhesion, biofilm formation, and toxin production (Hussain et al., 2022); tannins are capable to inhibit bacterial adhesion to epithelial cells, frequently considered as a significant step in GI infections pathogenesis (Piazza et al., 2023). Other polyphenols, such as anthocyanins and chlorogenic acid, control MDR-GI infections by regulating intestinal microbiota by inhibiting the colonization by pathogenic bacteria (Verediano et al., 2021; Bao et al., 2020), promoting autophagy of pathogenic bacteria (Tan et al., 2020), improving intestinal barrier function by increasing tight junction proteins and goblet cells (Verediano et al., 2021), and modulating the inflammatory responses by macrophages (Rathinasabapathy et al., 2022) (**Figure 2**).

Flavonoids, such as quercetin and naringenin, inhibit bacterial enzymes involved in DNA replication and protein synthesis, leading to reduced bacterial proliferation (Pathak and Mazumder, 2024). Other flavonoids, such as grape seed oligomeric proanthocyanidins, quercetin, and luteolin, alleviate oxidative stress, reduce inflammation, and damage the barrier integrity of infected cells (Kovács et al., 2024; Karancsi et al., 2022; Kovács et al., 2022; Zhang et al., 2020) (**Figure 2**).

Essential oils, such as thymol (extracted from thyme) or carvacrol (extracted from oregano), have been reported to exhibit antimicrobial activity by disrupting bacterial cell membranes, impairing ATP production, and interfering with quorum sensing, ultimately resulting in bacterial death (Trifan et al., 2020). Moreover, thymol and carvacrol protect intestinal epithelial cells by promoting cell integrity (Giovagnoni et al., 2020) (**Figure 2**).

Besides these three groups, other natural compounds, such as allicin and berberine, can also be used to manage MDR-GI infections. For instance, allicin, a sulfur-containing compound extracted from garlic (*Allium sativum*), has demonstrated efficacy against a broad spectrum of MDR-GI bacteria, including those responsible for biofilm formation, through its ability to disrupt bacterial membranes and inhibit quorum sensing (Bhatwalkar et al., 2021). Also, berberine, an alkaloid extracted from *Berberis* species, targets bacterial DNA replication and energy metabolism, showing potent activity against resistant strains of *E. coli* and *Salmonella* spp (Zhou et al., 2023).

Natural compounds combat MDR-GI pathogens through mechanisms distinct from conventional antibiotics, often bypassing common resistance pathways. These mechanisms include bacterial cell wall and membrane disruption, biofilm formation inhibition, and quorum sensing communication interference (Álvarez-Martínez et al., 2020b). For instance, allicin and essential oils, can integrate into bacterial lipid bilayers, causing membrane destabilization, leakage of cellular contents, and eventually bacterial lysis (Kachur and Suntres, 2020; Bhatwalkar et al., 2021). These effects are particularly effective against Gram-positive bacteria, which have a peptidoglycan outer cell wall, but also extend to some Gram-negative pathogens (Tavares et al., 2020).

Another target of natural compounds is biofilm formation, a primary defense mechanism of bacteria against antibiotics, which is complicated infection treatment (Damyanova et al., 2024). For instance, curcumin and allicin inhibit the production of extracellular polymeric substances (EPS) that constitute the structural matrix of biofilms, reducing bacteria’s ability to establish and maintain these protective environments (Pinto et al., 2020). Moreover, some natural compounds can penetrate biofilms, sensitizing bacteria to natural and synthetic antimicrobial agents (Sakarikou et al., 2020).

The last mechanism is associated with interference with bacterial quorum sensing, the communication system that regulates bacterial virulence, a novel mechanism offered by natural compounds (Yang et al., 2020). For instance, flavonoids (e.g., quercetin) and berberine can disrupt quorum sensing pathways by inhibiting the synthesis or activity of signaling molecules such as acyl-homoserine lactones (Deryabin et al., 2019). By interfering with quorum sensing, these compounds reduce bacterial coordination, virulence, and biofilm production and can be regarded as potential therapeutic approaches against MDR-GI bacteria (Bouyahya et al., 2022).

In treating *H. pylori* infections, natural compounds have been proven as particularly promising (Deng et al., 2024). For instance, curcumin inhibits bacterial adhesion to gastric epithelial cells, reduces inflammation, and suppresses virulence factors (Mohammadi et al., 2022). Allicin targets *H. pylori* biofilms and disrupts quorum sensing communication, offering an effective alternative for patients colonized by antibiotic-resistant strains (Sathianarayanan et al., 2022). Berberine, often used combined with conventional antibiotic therapies, has been shown to improve treatment outcomes for *H. pylori* by enhancing antibiotic activity and mitigating drug resistance (Hu et al., 2020).

Notably, the synergistic effects between natural compounds and antibiotics are another area of significant clinical interest (Álvarez-Martínez et al., 2020b). For instance, berberine enhances the activity of β-lactam antibiotics against *E. coli* (Zhou et al., 2023), while curcumin potentiates metronidazole against *C. difficile* and *H. pylori* (Mohammadi et al., 2022). Moreover, phytochemical compounds isolated from *Phyllanthus emblica*, demonstrated synergistic activity with ciprofloxacin and tetracycline against *Salmonella Typhimurium*, potentially by inhibiting efflux pumps (Mehta et al., 2016). Combinations of tetracycline with alkaloid-related compounds such as nitroxoline, sanguinarine, and zinc pyrithione showed synergistic effects against various diarrheic bacteria, including Shigella flexneri and E. coli (Osei-Owusu et al., 2024). Phenylpropanoids and flavonoids, particularly luteolin and sinapic acid, enhanced the efficacy of ciprofloxacin and gentamicin against *S. aureus*, *Klebsiella pneumoniae*, and *P. aeruginosa* (Kincses et al., 2024). These combinations allow for lower doses of antibiotics, reducing side effects and toxicity and potentially overcoming bacterial resistance mechanisms (Xiao et al., 2023).

Natural compounds promise to treat gastrointestinal infections, but several challenges hinder their clinical translation. Recent studies (Qassadi et al., 2023) highlight the therapeutic potential of plant-derived products against gastrointestinal pathogens, emphasizing the need for quality assessments and research gaps in their efficacy. Others (Murphy et al., 2020) identify β-glucans’ diverse therapeutic properties and the complexities in their mechanisms of action, which complicate clinical testing. They also highlight the variability in β-glucan preparations due to differences in sources and extraction methods. These studies underscore the need for further research to overcome these challenges in natural compound translation.

Other challenges that limit their widespread application in clinical practice (Heinrich et al., 2020) include its variability in bioavailability and potency (Borges et al., 2020). Some natural compounds, such as curcumin and berberine, present low solubility and poor absorption in the GI tract, often resulting in suboptimal systemic concentrations, which are insufficient to adequately treat MDR-GI infections (Hu et al., 2023). Advances in delivery systems, such as nanoformulations (e.g., liposomes, polymeric nanoparticles, and micelles) (Borges et al., 2020) and encapsulation technologies (e.g., spray drying, liposomes, emulsions, and nanoencapsulation) (MaChado et al., 2019), are currently being developed to enhance natural compounds’ bioavailability and therapeutic efficacy (**Figure 3**).

Other matters of concern include the potential adverse effects of high doses of natural compounds and interactions between co-administrated drugs (Malczak and Gajda, 2023). As in conventional medicines, high doses of natural compounds can also be associated with adverse effects in some patients. The deleterious effects are gastrointestinal irritation, hepatotoxicity, and allergic reactions (Vilas-Boas et al., 2021). Additionally, natural compounds can interact with conventional, altering their metabolism and effects during infection. For instance, berberine binds to cytochrome P450 enzymes (e.g., CYP3A4, CYP2D6, CYP2C9, CYP2C19), preventing other drugs from being metabolized, resulting in higher plasma concentration of the co-administrated drug and increasing the risk of toxicity (Zhang et al., 2021) (**Figure 3**).

As in the case of probiotics, the lack of standardization and regulatory guidelines also delays the clinical application of natural compounds (Andreani et al., 2024). Variability in the composition of plant-derived products is due to differences in cultivation, extraction, and storage methods. This variability can lead to unpredicted efficiency, making it difficult to ensure consistent therapeutical benefits and conflicting results or irreproducible findings during clinical trials (Scotti et al., 2019). Moreover, the lack of standardized protocols for safety and efficacy and the limited regulatory oversight undoubtedly hinders the integration of natural compounds into formal treatment guidelines (Komala et al., 2023) (**Figure 3**).

## 5 Bacteriophage

Bacteriophage (or phage) can be considered an alternative therapeutical approach for MDR-GI infections (Gutierrez and Domingo-Calap, 2020). Bacteriophages, defined as viruses that infect and lyse bacteria with remarkable specificity (i.e., targeting only harmful bacteria while sparing beneficial ones), offer unique advantages to combat these infections and present distinct action mechanisms to conventional drugs (Kwiatek et al., 2020). Bacteriophages can be administered through various routes, such as topical application, inhalation, oral, or parenteral delivery (Düzgüneş et al., 2021).

One of the primary bacteriophage action mechanisms is the specific targeting and lysis of bacterial pathogens. Bacteriophages recognize and bind to specific receptors on the surface of the targeted bacteria and transfer their genetic material into the cell. Once inside, the bacteriophage hijacks the bacterial machinery and produces new viral particles. At the end of this process, bacteria undergo a lysis process, and the new bacteriophage progeny is release (Dunne et al., 2021). The specificity in bacteria-bacteriophage recognition guarantees that only the targeted bacterial pathogens are affected, leaving the commensal microbes from the normal gut microbiota intact. This aspect constitutes a critical advantage of bacteriophages compared to conventional broad-spectrum antibiotics (**Figure 2**).

Another significant mechanism is the inhibition of bacterial biofilm production. Biofilms are structured microorganism communities enclosed by a self-produced extracellular polymeric substance (EPS) that adheres to surfaces and protects bacteria from the action of antibiotics and the immune system, leading to persistent and chronic infections (Zafer et al., 2024). Bacteriophages can penetrate these biofilms and enter inside the bacterial cells, replicate and lead to their destruction, preventing infection dissemination (Singh et al., 2022). Additionally, specific bacteriophages also produce enzymes, such as depolymerases and polysaccharide lyases, which degrade specific structures within the biofilm matrix facilitating bacteriophage access to target bacteria (Topka-Bielecka et al., 2021). This dual action not only destroys bacteria within biofilms but also disrupts the structural integrity of the biofilm itself, making it more susceptible to other therapeutic agents (Talapko and Škrlec, 2020) (**Figure 2**).

The clinical evidence supporting the efficiency of bacteriophage therapy continues to grow, with several successful cases documented (Luong et al., 2020). For instance, preclinical trials have demonstrated that bacteriophages targeting *C. difficile* reduce bacterial colonization and toxin production (Nale et al., 2022. Similarly, in *Salmonella* infections in animal models, bacteriophages’ ability has been demonstrated to mitigate symptoms and limit bacterial colonization (Kosznik-Kwaśnicka et al., 2022; Thanki et al., 2022). Human clinical trials and case studies have also provided promising results, even in patients with a severe *E. coli* infection unresponsive to antibiotics (Broncano-Lavado et al., 2021. Ongoing trials evaluating bacteriophage in *C. difficile* and *Salmonella* spp. infections have shown significant reductions in bacterial load and improvements in symptoms, with minimal side effects (Heuler et al., 2021; Lamy-Besnier et al., 2021).

Bacteriophage therapy offers several compelling advantages. One of the most significant is its high specificity. Contrary to conventional antibiotic therapeutics that indiscriminately target several bacteria, bacteriophages act exclusively on their bacterial host (Kwiatek et al., 2020). Bacteriophage specificity minimizes the collateral damage on the gut’s commensal microbiota, preserving its essential functions and reducing the risk of secondary infections such as *C. difficile*-associated diarrhea (Nale et al., 2022). Also, bacteriophages have a minimal impact on the host’s overall microbiome, maintaining microbial diversity and stability often disrupted by conventional antibiotic therapy (Mu et al., 2021).

While phage therapy shows promise in clinical case studies and randomized controlled trials, several challenges hinder its widespread adoption (Petrovic Fabijan et al., 2023). These include bacterial strain variation, phage resistance, and potential limitations of host immune responses (Hatfull et al., 2022). Safety profiles appear favorable, with mild to moderate adverse events reported in only 5.1% of participants in recent trials (Walter et al., 2024). However, standardization of protocols, including phage preparation quality, sensitivity testing, and dosage optimization, is crucial for successful clinical translation (Walter et al., 2024) (**Figure 3**).

Regulatory constraints remain a significant obstacle. Contrarily to traditional drugs, bacteriophages are living microorganisms capable of evolving, an aspect that further complicates their regulatory approval (Brives and Pourraz, 2020). Therefore, each batch of new bacteriophage preparations must be rigorously evaluated in terms of safety, efficacy, and quality, a resource-intensive and time-consuming process (Verbeken and Pirnay, 2022). Noteworthy, bacteria can also develop resistance to bacteriophages, often involving mutations in the receptors that bacteriophages use to recognize target bacteria. For instance, *Salmonella enterica* bacteriophage-resistant mutants exhibited increased tetracycline susceptibility and decreased virulence, with mutations in lipopolysaccharide genes (Fong et al., 2020). Extraintestinal pathogenic *E. coli* developed resistance through mutations in lipopolysaccharide biosynthesis and outer membrane genes, resulting in attenuated virulence (Salazar et al., 2021). However, bacteria resistance to bacteriophages can be mitigated by using bacteriophage cocktails with broad host ranges targeting different bacterial receptors (Vaitekenas et al., 2021) or by engineering bacteriophages to overcome bacterial defenses (Bleriot et al., 2024) (**Figure 3**).

Another critical challenge is the bacteriophage production and standardization. Producing bacteriophages at a clinical scale requires rigorous quality control to ensure their purity, stability, and efficiency (João et al., 2021). The diversity of bacteriophages and the need for strain-specific formulations further complicate standardization procedures (Nale et al., 2022). Developing scalable and reproducible manufacturing processes, including optimizing fermentation, purification, formulation processes, and quality control, will bring bacteriophage therapy into clinical practice (Malik, 2021) (**Figure 3**).

## 6 Integrative approaches

Multidrug-resistant gastrointestinal infections constitute a serious worldwide health problem that constantly demand alternative therapeutic strategies that rise above conventional antibiotic therapies. One solution to this problem is integrative approaches combining the benefits of probiotics, natural compounds, and bacteriophage. This approach has great potential in enhancing treatment efficacy and minimizing the development of resistance since lower antibiotic dosage can be applied with the same treatment outcomes (Ghosh et al., 2019). Another solution is the emerging field of personalized medicine that offers tailored therapies adjusted according to the unique composition of the microbiome and clinical context of each patient (Bhat et al., 2022). Microbiome-targeted interventions, such as fecal microbiota transplantation, probiotics, and microbiota-targeted diets, are also being explored to enhance treatment outcomes (Liwinski and Elinav, 2020).

Combination therapies to manage MDR-GI infections, involving the synergistic use of multiple therapeutic agents, contribute to better outcomes when compared with each of those applied individually (Cheng et al., 2019; Garvey, 2020). By combining the complementary effects of probiotics, natural compounds, and bacteriophages, integrative approaches can significantly enhance efficacy, mitigate resistance, and restore microbiome homeostasis (Newman and Arshad, 2020). Through competitive inhibition, immune modulation, and gut barrier reinforcement, probiotics can suppress pathogen growth and facilitate microbiome recovery (Peters et al., 2019; Rueda-Robles et al., 2022; Tasaka et al., 2023). Natural compounds target bacterial membranes, biofilm formation and stability, and quorum sensing, weakening pathogens and enhancing their susceptibility to other therapies (Álvarez-Martínez et al., 2020b; Song et al., 2021; Panda et al., 2022). Bacteriophages can directly target resistant pathogens with their specific bacterial lytic activity and biofilm-disrupting enzymes (Liu et al., 2022). When applied together, these therapies create a complex attack on MDR-GI infections, improving treatment outcomes. For instance, bacteriophages can reduce the population of resistant pathogens due to their biofilm-degrading enzymes, rendering bacteria more susceptible to probiotics and natural compounds; probiotics can re-colonize the GI tract and restore microbiome homeostasis more effectively; finally, natural compounds enhance bacteriophage efficacy by weakening bacterial defenses. This synergistic interaction among alternative therapeutical approaches improves pathogen eradication and promotes gut homeostasis, reducing the likelihood of reinfection and drug resistance development.

The complexity of MDR-GI infections requires personalized therapies considering the individual microbiota composition variability, host immune responses, and the nature of the infection. The personalized medicine approach aims to tailor treatments to patients’ unique characteristics, optimizing outcomes and minimizing its adverse effects (Salih et al., 2023). The human gut microbiome is highly characteristic of a given individual, presenting variations in composition and metabolic activities. These variations influence host susceptibility to bacterial infections and treatment responses (Jain, 2020). The recent advances in metagenomic sequencing and microbiome analysis allow for more detailed identification of specific disease-associated dysbiosis patterns and pathogenic strains (Mousa et al., 2022). Metagenomic analysis can also assist clinicians in selecting the most effective probiotics, natural compounds, and bacteriophages for a given patient according to the microbiome profile. For instance, patients with reduced populations of commensal bacteria (e.g., *Lactobacillus* or *Bifidobacterium*) benefit from targeted probiotic supplementation to restore microbiota homeostasis. Bacteriophages specific to those pathogens can be incorporated into patients where pathogenic species dominate. Additionally, natural compounds can be selected based on their activity spectrum to target specific bacterial defenses.

Since bacteria can manipulate host epigenetic mechanisms to enhance their survival, these personalized treatments should also consider host factors, such as immune status, comorbidities, and genetic predispositions (Crimi et al., 2020). For instance, in immunocompromised patients, probiotics’ safety should be carefully evaluated to avoid potential complications, such as bacteremia and sepsis (Mikucka et al., 2022). Similarly, natural compounds’ pharmacokinetic and pharmacodynamics may vary based on the patient’s metabolic and absorptive capacity, requiring individualized dosing or alternative delivery systems (e.g., lipid-based nanoparticles, polymeric nanoparticles, and self-microemulsifying systems) that enhance bioavailability, improve stability, and allow for controlled release of the selected natural compounds (Patel et al., 2024; Grilc et al., 2021).

Finally, the infection specifics (e.g., location, severity of GI involvement, and clinical presentation) also play an important role in treatment design. For instance, localized infections in the small intestine require therapies that can effectively deliver into this region (e.g., encapsulated bacteriophages or targeted probiotic strains). In contrast, systemic complications, such as bacteremia arising from gut pathogens, require adjunctive systemic therapies alongside localized interventions. Imaging techniques (e.g., Computed Tomography, Positron Emission Tomography, Ultrasound, and Magnetic Resonance Imaging) play an important role in differentiating between various inflammatory and infectious conditions in the GI tract (Frickenstein et al., 2019), allowing clinicians to formulate a better diagnosis and suggest a suitable treatment protocol.

## 7 Tailoring non-antibiotic therapies for MDR-GI infections: challenges and innovations in bacteriophage and probiotic treatments

The efficacy of the proposed non-antibiotic treatments varies according to bacterial strains and patient populations. Bacteriophages are typically strain-specific, meaning that a phage cocktail that combats one *E. coli* strain may fail against another due to differences in surface receptors or resistance mechanisms (Korf et al., 2020). While oral phage therapy showed promise in reducing *E. coli* infections in piglets (Mao et al., 2023), its efficacy in chickens was limited (Kittler et al., 2020). In a murine model, phage therapy demonstrated only transient reduction of multidrug-resistant *E. coli* intestinal carriage (Javaudin et al., 2021). In a trial using oral phage therapy for pediatric diarrhea in Bangladesh, the treatment did not significantly improve the outcomes, probably due to insufficient coverage of the phage preparations of the *E. coli* strains, only achieving minimal replication in the gut (Sarker et al., 2016). These results reinforce the challenges in achieving consistent efficacy with phage therapy, particularly in reducing bacterial digestive carriage *in vivo* (Marongiu et al., 2022).

Patient-to-patient variability also influences treatment outcomes. Differences in individual gut microbiome composition, physiology, and diet may alter how these therapies perform (Feng et al., 2020). Moreover, a patient’s immune system may neutralize or clear therapeutic viruses or bacteria: repeated phage dosing can induce anti-phage antibodies or phagocyte uptake that reduces phage bioavailability over time (Berkson et al., 2024). Gastric pH and motility also affect orally delivered therapies (Dass et al., 2025). For instance, oral phages and probiotic bacteria must survive stomach acidity to reach the intestine, and variations in these factors between individuals can lead to inconsistent results (Bernatek et al., 2022).

Advanced diagnostics are nowadays employed to improve results’ reproducibility. Rapid genomic sequencing of the pathogen identifies its resistance genes and surface antigen profile, directing the choice of the most suitable matching bacteriophage (Liu et al., 2022). In bacteriophage therapy, creating a phage library and doing *in vitro* host-range testing on the patient’s bacterial isolate (similar to an antibiotic susceptibility test) can ensure that the selected phages will infect the specific strain colonizing the host (Bozidis et al, 2024). Furthermore, emerging approaches such as personalized bacteriotherapy tailor probiotic or phage cocktails to an individual’s microbiome and infection profile (Ferry et al., 2022; Köhler et al., 2023). These strategies and standardized manufacturing of natural compounds (to minimize batch variability in plant-derived antimicrobials) reduce the variability in treatment effectiveness across diverse populations (Vaou et al., 2021).

Growing clinical evidence is being gathered for these alternative therapies, especially in the context of drug-resistant GI infections. Bacteriophage therapy is being tested in controlled trials after many decades of case-based use. A recent milestone is the Phase 1 trial of SNIPR001, a CRISPR-enhanced phage cocktail targeting E. coli in the gut [refs]. In this study (36 healthy volunteers and patients), oral SNIPR001 for 7 days was well tolerated and significantly reduced gut E. coli levels, including antibiotic-resistant strains, demonstrating that engineered phages can safely modulate the GI microbiota and potentially prevent MDR infections in high-risk hosts (e.g. stem-cell transplant patients prone to *E. coli.* bacteremia) (Paterson, 2024).

Other trials have focused on compassionate use. For instance, case series have reported successful bacteriophage treatment of chronic *Salmonella* and *Shigella colitis* or decolonization of MDR *Klebsiella* in the gut (Emencheta et al., 2023; Mohammadi et al., 2023; Fang et al., 2024). However, data from randomized controlled trials are still limited (Górski et al., 2020).

Ongoing trials (as listed on ClinicalTrials.gov) evaluate phages for recurrent *C. difficile* (using bacteriophage or bacteriophage-derived lysins) and decolonizing Carbapenem-resistant Enterobacterales (CRE) in intensive care patients. Regulatory authorities in some regions (e.g., Belgium and France) have also initiated phage therapy programs, enabling clinical access while gathering efficacy data.

Probiotic interventions have been examined, although fewer are explicitly targeted at MDR pathogens. Some trials have used probiotics such as *Lactobacillus rhamnosus* GG and *Saccharomyces boulardii* to prevent recurrent *C. difficile* or to suppress Vancomycin-resistant Enterococcus (VRE) colonization, with mixed results (modest reductions in recurrence or colonization, but not complete eradication) (Rahman et al., 2024). A novel approach in development is the use of genetically engineered probiotic strains to deliver antimicrobial effectors. For example, an *E. coli* Nissle 1917 probiotic engineered to produce a bacteriocin (microcin MccI47) has been proven effective against carbapenem-resistant *Klebsiella pneumoniae* (Sassone-Corsi et al., 2016). In a pre-clinical mouse model, this engineered probiotic significantly reduced gut colonization by carbapenem-resistant *K. pneumoniae* (KPC) compared to controls (Mortzfeld et al., 2022). Another study identified microcin MccM as the primary antibacterial agent in EcN against *Salmonella*, with overexpression of MccM significantly reducing pathogen adhesion and invasion in intestinal epithelial cells (Ma et al., 2023).

While not yet in human trials, this illustrates the live biotherapeutic product designed to target MDR-GI pathogens *in situ*. Such interventions are expected to enter clinical trials shortly as live biotherapeutics (with one example being a Phase 1 study of an oral *Enterococcus* probiotic to reduce VRE colonization).

Additionally, various natural compounds (e.g., plant-derived polyphenols and essential oils) with antimicrobial and anti-biofilm activity are being evaluated preclinically and in small trials for GI pathogens. For instance, certain alkaloids, terpenoids, and sulfur-containing phytochemicals have been proven as exhibiting antibacterial properties against various GI pathogens, such as *E. coli*, *C. difficile*, *Campylobacter* spp., and *Salmonella* spp., but their clinical efficacy and safety is still under investigation (Qassadi et al., 2023).

Clinical trials indicate promising roles for non-antibiotic therapies in MDR-GI infection management but also highlight variability in outcomes and the need for optimized protocols. Regulatory considerations are important in trial design. For instance, bacteriophage production must be performed under highly quality-controlled conditions, even in experimental settings. Ongoing study results will clarify safety and efficacy, guiding eventual approvals and clinical use.

Broad-scale implementation of these non-antibiotic therapies is often associated with significant financial and logistical considerations. Production costs for these biological therapies can be elevated. Bacteriophages require fermentation and purification under Good Manufacturing Practice (GMP) standards if used as medicinal products (Bretaudeau et al., 2020). Currently, GMP manufacture of a batch of therapeutic phage cocktails (for a few hundred doses) can exceed £500,000, reflecting the expenses of quality control and the lack of economies of scale (Suleman et al., 2024). Also, as naturally occurring phages cannot be patented, pharmaceutical investment has been limited, slowing industrial development, limiting economic incentives to streamline production, and keeping costs relatively high (Todd, 2019).

Many of these therapies require highly specialized infrastructure that may be lacking in resource-limited countries (Khalid et al., 2021). Bacteriophage therapy may require laboratory support to isolate and test phages for each patient (if personalized) and appropriate formulation (e.g. buffer or encapsulation to survive gastric transit). Maintaining a bacteriophage library to access many bacteriophages is a proposed solution in high-income healthcare systems (Nagel et al., 2022). Establishing and maintaining these libraries requires investment in microbial labs, acquiring phage collections, and data management. Probiotic production at scale also requires fermenters and quality testing to ensure stability and purity, excluding any unwanted microbial contamination (Zawistowska-Rojek et al., 2022).

In low-resource countries, these factors constitute significant challenges. However, there is potential for local manufacturing and sourcing to improve feasibility. Countries such as Georgia, Poland, and Russia locally produce phage cocktails, enabling continued phage therapy use at a relatively low cost outside Western regulatory frameworks (Ferry et al., 2021). Establishing regional phage production centers reduces the dependence on imported products. Local phage isolation against endemic MDR strains – essentially creating region-specific phage libraries – improves relevance and reduces the costs of shipping and licensing external phages (Nagel et al., 2022). Similarly, naturally derived compounds (e.g., plant extracts) could be sourced and processed regionally to treat GI infections, though batch-to-batch consistency must be addressed (Qassadi et al., 2023). Community-based initiatives and local R&D investment are key element that will allow to overcoming cost barriers in LMICs.

Another aspect of cost is the health-economic impact. While the upfront costs of these alternative therapies are high, they can be considered cost-effective as they avoid prolonged hospitalizations and the use of last-line antibiotics (Gupta and Ananthakrishnan, 2021).

Therefore, the scalability of non-antibiotic MDR-GI therapies dramatically depends on the healthcare setting. High-income countries are moving toward industrial-scale production. In contrast, LMCIs might focus on simpler delivery forms and local resource utilization. Strategic investments, technology transfer, and international partnerships (e.g., supporting a GMP phage facility that serves multiple hospitals or countries) are potential ways to broaden access. Technological improvements (e.g., more efficient phage fermentation, synthetic biology to produce antimicrobial compounds in less expensive hosts, and improved shelf-stable formulations reducing logistic complexity) may lead to cost reduction (Malik, 2021).

All therapeutic interventions are associated with health risks, and non-antibiotic treatments are no exception. Potential risks include off-target effects, unintended ecological disturbances, the development of resistance against the therapy, and various safety concerns (Liu et al., 2021).

One advantage often cited for bacteriophage and probiotic therapy is their targeted action and sparing of the normal microbiota, due to their highly specific bacteriophages act on target bacterium and leaving other species unharmed (Zhang et al., 2021). Although rare, off-target impacts may be reported. For instance, broad-spectrum bacteriophages or lytic enzymes might inadvertently attack beneficial commensal strains due to shared targeted receptors (Łobocka et al., 2021). Similarly, introducing a live microbe (probiotic) can unpredictably alter microbiome composition, potentially suppressing some native species (Mousa et al., 2023). To mitigate these off-target effects, phage cocktails are selected to narrow the host range to pathogens of interest. Also, the patient’s microbiome is often monitored before and after therapy towing to identify any changes and to recommend interventions such as targeted probiotics prescription to restore balance (Terwilliger et al., 2021; Hatfull et al., 2022).

As bacteria develop resistance to antibiotics, they can evolve resistance to bacteriophages, bacteriocins, or other biotherapies. Bacteria exposed to phage may mutate the phage receptor or activate defense systems (e.g. CRISPR, restriction-modification) that render the phage ineffective (Hasan & Ahn, 2022; Costa et al., 2024).

There is a growing concern that bacteriophage overuse may result in broadly phage-resistant bacterial strains, which might then spread into the environment (Hassan et al., 2021). To minimize this risk, cocktails containing multiple phages targeting different bacterial receptors are employed so that a single mutation will not confer resistance to all components (Borin et al., 2023). Moreover, bacteriophages can be engineered or “trained” through experimental evolution to overcome bacterial defense mechanisms (Borin et al., 2023). Rotating or adjusting therapeutic cocktails based on genomic surveillance of the pathogen can also help. Notably, as these therapies often do not exert the same broad selective pressure as antibiotics, the hope is that they may impose less long-term evolutionary pressure for resistance in the overall microbial community (Merker et al., 2020). Nonetheless, stewardship of bacteriophage and probiotics (avoiding unnecessary exposure) is advised to slow resistance development.

There is a risk of transmitting harmful organisms or causing infection in the host, particularly for live biotherapeutics such as probiotics. Probiotic products must be free of contaminants; there have been rare reports of probiotics causing bloodstream infections in immunocompromised patients, so caution is used in those populations. If not appropriately purified, bacteriophage preparations could introduce endotoxins or bacterial DNA from their production cultures. Modern bacteriophage therapy efforts use high-purity preparations and often administer bacteriophages initially at low doses to observe any inflammatory reaction before escalating the dose. Thus far, phage therapy has shown a favorable safety profile, with most reported adverse events mild and often attributable to underlying illness rather than the phage itself (Liu et al., 2021). Still, oversight is needed – for example, avoiding phages that carry toxin genes or can transduce bacterial DNA. Whole-genome sequencing of therapeutic phages is standard to ensure they do not harbor virulence or antibiotic resistance genes.

Altering the gut ecosystem (dysbiosis) could have unintended long-term consequences. Successful decolonization of an MDR organism via bacteriophage might open a niche that another organism fills. For instance, if phage therapy eliminates all *E. coli* in the gut (as SNIPR001 is designed to do), one must consider what replaces that population – possibly other Gram-negative rods like *Klebsiella* or *Proteus*, which could be problematic if they overgrow (Gencay et al., 2024). In the Bangladeshi phage trial, researchers noted that treated children’s microbiota were dominated by *Streptococcus* species during acute diarrhea. However, this was also seen in placebo patients and may reflect the disease state rather than the phage action (Marongiu et al., 2022). To mitigate dysbiosis risks, therapies are often paired with measures to support a healthy microbiome. For example, some protocols suggest giving a probiotic after bacteriophage or antibiotic therapy to restore microbial diversity (Mu et al., 2021). When using potent natural antibacterial compounds, sublethal dosing is avoided to prevent wiping out too much of the flora (Pagnossa et al., 2021). Finally, long-term surveillance of patients who receive these therapies (through registries and follow-up studies) is important to detect any late-emerging issues such as new-onset autoimmune conditions or metabolic changes, which are theorized but not proven risks of microbiome manipulation (Merenstein et al., 2023).

Therefore, risk mitigation implies rigorous clinical protocols and monitoring (Doub, 2021). Regulatory agencies have issued guidelines (e.g., bacteriophage IND requirements). Researchers are devising safeguards such as destroying switches in engineered microbes and refining delivery systems (e.g., encapsulating bacteriophages to release in the colon, reducing systemic exposure) (Doub, 2021). By learning from early experiences and adverse events, the safety profile of these non-antibiotic therapies continues to improve, making them more viable for broader use.

Integrating non-antibiotic therapies into mainstream clinical practice and antimicrobial stewardship frameworks requires careful planning but offers a valuable opportunity to enhance patient care while reducing reliance on traditional antibiotics (Maillard et al., 2021). To include therapies such as bacteriophages, probiotics or natural compounds into existing regimens, clinicians demand clear guidelines on when and how to use them. For example, a phage therapy consult might be recommended for a persistent MDR *Salmonella* or decolonizing a high-risk CRE carrier after antibiotic therapy (Suh et al., 2022). Early integration may involve using these therapies as adjuncts. In practice, a patient with a severe MDR-GI infection might receive conventional antibiotics to stabilize acute illness but then a non-antibiotic therapy to eradicate residual pathogens or prevent relapse (Liu et al., 2021). Combination therapy can be synergistic; studies have shown that using phages with antibiotics can sometimes enhance bacterial clearance more than alone (Łusiak-Szelachowska et al., 2022).

Thus, stewardship programs can incorporate protocols for adjunctive phage or probiotic use in defined scenarios (e.g. adjunct bacteriophage for an MDR infection responding poorly to antibiotics). This combined approach must be balanced to ensure the non-antibiotic alternatives do not interfere with antibiotic action (or vice versa). However, many bacteriophages are compatible with certain antibiotics and even protective of gut flora during antibiotic treatment (Liu et al., 2022; Łusiak-Szelachowska et al., 2022).

From an antimicrobial stewardship perspective, the judicious use of non-antibiotic therapies can help preserve antibiotic efficacy (Łusiak-Szelachowska et al., 2022). Whenever a bacteriophage cocktail successfully treats an infection that would otherwise require a last-resort antibiotic, it spares that antibiotic from use, slowing the spread of resistance genes. Stewardship programs can broaden their scope to include these alternatives as tools to reduce antibiotic consumption. However, such use must be evidence-based. Programs will need to track the outcomes of these therapies and ensure they are used in appropriate patients (much as stewardship tracks appropriate vs. inappropriate antibiotic use). Since non-antibiotic alternatives are relatively novel, one challenge is educating clinicians – infectiologists and pharmacists will need training on indications and handling of these therapies. Multidisciplinary stewardship teams may start to include microbiome specialists or phage therapy experts who can advise on cases.

Regulatory acceptance is a significant factor in integration. In some countries, regulations have evolved. In most countries, integration is still at an earlier stage since bacteriophages are available only under compassionate use or experimental IND protocols (Yang et al., 2023). However, initiatives like the Belgian magistral bacteriophage framework (where pharmacies can prepare patient-specific phage cocktails on prescription) and the UK’s planned national phage library (Verbeken and Pirnay, 2022) point to possible models for broader availability. Regulatory agencies must update guidelines to accommodate the unique nature of phages (e.g. allowing for adaptable formulations). This is an ongoing challenge – for instance, defining bacteriophage therapy as a drug vs. a biologic and determining requirements for approval are still being debated internationally. Overcoming these hurdles is key to moving phages from last-resort exceptions to regular options integrated into care.

Ensuring consistent quality and outcomes is essential when integrating new therapies. Hospitals implementing bacteriophage therapy should have oversight committees such as pharmacy and therapeutics committees for medications. They must maintain quality control (e.g., verifying phage titers and the absence of contaminants in each batch). Moreover, outcome monitoring (did the therapy achieve cure or decolonization? Were there any adverse events)? should be fed back into clinical practice improvements. Over time, this will build a knowledge base that refines patient selection criteria – identifying who benefits most from these therapies.

Ultimately, integration into practice will likely involve combination and personalization. Recent perspectives noted that synergistic use of probiotics, bacteriophages, and natural compounds could yield the best outcomes while minimizing resistance development. For example, a “microbiome therapy bundle” might include an initial suppressive treatment (bacteriophage or natural compounds) followed by a restorative treatment (probiotic) to reset the gut ecology. Personalized medicine approaches could tailor this bundle: a patient’s microbiome and pathogen genomics might be analyzed to choose the optimal bacteriophage and probiotic species for that individual. Antimicrobial stewardship programs will expand to microbial stewardship, managing drug prescriptions and microbiome health (Watkins, 2022).

Although incorporating non-antibiotic therapies into standard care for MDR-GI infections constitutes a promising strategy to improve outcomes and curb antibiotic resistance, it requires overcoming regulatory barriers, proving cost-effectiveness, and educating healthcare providers. As more clinical trial data emerge and success stories accumulate, these alternative therapies move from experimental options to components of evidence-backed treatment guidelines. With appropriate stewardship, they can complement antibiotics – using the right tool for the right infection – thereby advancing a more sustainable and precise approach to managing complicated gastrointestinal infections,

## 8 Future directions and research needs

Although non-antibiotic alternatives, such as probiotics, natural compounds, and bacteriophages, show promise in combating MDR-GI infections, future research should address key challenges before these therapies are more widely applied in clinical practice.

One key challenge that should be further studied is the protocols for combination therapies, one of the most promising approaches for treating MDR-GI infections (Gray and Wenzel, 2020). Combining probiotics, natural compounds, and bacteriophage therapies makes it possible to profit from the synergistic effects of all approaches in terms of enhanced treatment efficacy and minimized resistance development. Therefore, future research should focus on optimizing these combinations to achieve maximal synergy. This will be achieved by identifying the most compatible combinations between probiotics, natural compounds, and bacteriophages for specific pathogens and infection contexts.

Another challenge is personalized medicine, which aims to tailor therapeutic interventions based on the unique characteristics of individual patients (Salih et al., 2023). It presents an immense potential for MDR-GI infection treatment. According to this principle, variations in microbiome composition, host immune response, and infection dynamics alter the treatments’ outcomes and must be finely adjusted to provide optimum results. Metagenomic sequencing and microbiome analysis advances have identified dysbiotic patterns and specific pathogens within the gut microbiome (Mousa et al., 2022). This information guides clinicians to select probiotics, natural compounds, and bacteriophages most effective against the identified pathogens. Host factors, such as immune status, genetic predispositions, and comorbidities should also be considered (Crimi et al., 2020). Immunocompromised patients may require alternative probiotics or bacteriophages with well-established safety profiles. Improved delivery systems (e.g., encapsulated probiotics or bacteriophages designed to target specific gut regions) will further refine treatment strategies. In the last decades, the role of the gut microbiome in health and disease has become a central research topic, with bidirectional interactions between microorganisms and medicines significantly impacting treatment outcomes (Weersma et al, 2020). Therefore, future studies should address the existing knowledge gaps on how probiotics, natural compounds, and bacteriophages influence microbiome composition and function. Understanding these interactions will allow the design of therapies that eradicate pathogens and promote long-term microbiome stability. Research should further explore the impact of microbiome diversity on treatment outcomes. For instance, if a diverse microbiome enhances gut ecosystem resilience, preventing pathogen overgrowth due to niche occupation and nutrient overlap, a depleted microbiome requires more intrusive interventions or prebiotic supplementation to support probiotic colonization (Dogra et al., 2020).

The third challenge is the development of effective delivery systems for therapeutic agents to the infection site. As such, future studies in delivery systems will undoubtedly enhance the efficacy of non-antibiotic therapies. Encapsulation technologies (e.g., alginate beads or lipid nanoparticles) should be developed to protect probiotics and bacteriophages from gastric acid and bile salts, ensuring their viability until they reach the infection site (Yao et al., 2020; Dlamini et al, 2023). Moreover, targeted delivery mechanisms (e.g., pH-responsive or enzyme-triggered capsules) should be developed to release the selected medicines precisely where they are needed and minimize off-target effects (Manzari-Tavakoli et al, 2024). Additionally, microfluidic platforms and bioengineered scaffolds should enable the co-delivery of the natural compounds, allowing the optimization of their combined effects and overcoming the limitations of traditional methods (Rosellini and Cascone, 2023; Teixeira et al., 2023).

Another challenge is associated with non-antibiotic therapy testing. While preclinical and small-scale trials have demonstrated the potential of non-antibiotic therapies to manage MDR-GI infections, large-scale clinical trials have failed to validate their efficacy and safety. Future trials should evaluate the outcomes of combination therapies in diverse patient populations and different MDR-GI infections. Standardized protocols for measuring clinical endpoints, such as pathogen eradication, microbiome restoration, and recurrence rates, should be prepared and discussed. Preclinical and clinical trials should investigate the long-term effects of non-antibiotic alternative therapies, including their impact on microbiome stability and resistance development. Comparative studies evaluating combination therapies against standard antibiotic treatments will provide valuable insights into their relative benefits and limitations.

Finally, the last challenge relates to the regulatory landscape for these non-antibiotic therapies that remain underdeveloped and constitute a significant obstacle to their more widespread adoption in clinical practice (Kumar et al, 2021). Probiotics, natural compounds, and bacteriophages face unique regulatory constraints mainly motivated by their biological complexity and variability. Therefore, regulatory agencies should contribute to pushing forward the use of alternative therapies by establishing precise safety, efficacy, and quality evaluation guidelines. Regarding bacteriophages, issues concerning bacteriophage-bacteria coevolution and strain specificity must be carefully considered (Piel et al., 2022). In the case of probiotics and natural compounds should undergo rigorous standardization to ensure consistent composition and efficiency. Collaborative efforts between researchers, the pharmaceutical industry, and regulatory agencies is quintessential to developing frameworks that facilitate the approval and commercialization of these non-antibiotic alternative therapies.

## 9 Conclusions

The rise in MDR-GI infections constitutes a significant concern for public health. To manage this worldwide problem innovative therapeutic approaches are required to surpass the challenges associated with traditional antibiotic treatments. Alternative non-antibiotic therapies should include probiotics, natural compounds, and bacteriophages, each offering unique action mechanisms, advantages, and challenges. Together, these alternative therapeutical approaches will allow the development of more effective, sustainable, and personalized interventions to combat MDR-GI infections.

It has been proven that probiotics inhibit MDR-GI bacteria, enhance the gut’s mucosal barrier, and modulate the immune response, effectively treating GI infections. However, several obstacles related to strain specificity, regulatory inconsistencies, and safety limited their clinical application, mainly in immunocompromised patients. Natural compounds also offer diverse antimicrobial mechanisms, such as membrane disruption, biofilm production inhibition, and quorum sensing interference. Nevertheless, despite their potential, their variability in bioavailability, potency, and standardization constitutes serious barriers to their widespread clinical use. Bacteriophage therapy is a powerful tool for fighting MDR-GI infections due to its precision targeting specific bacterial pathogens and biofilm disruption capabilities. However, bacterial resistance to bacteriophages, regulatory obstacles, and production challenges remain significant despite its advantages.

The effectiveness and reproducibility of the presented non-antibiotic therapies in managing MDR-GI infections exhibit considerable variability across different populations and microbial strains, which constitutes a significant challenge to their broad application in clinical settings. This variability includes pathogens’ genetic diversity, response to specific treatments, and differences in individuals’ gut microbiota composition, which influences the efficacy of microbial-based therapies like probiotics and bacteriophages. Additionally, environmental and dietary differences can alter the gut microbiome’s response to natural compounds, further complicating the predictability and reproducibility of treatment outcomes.

Clinical trials for alternative therapies address challenges such as the previously described variability in treatment effectiveness across different microbial populations and strains. Detailed and updated trial information, including statuses and results, can be accessed through databases like ClinicalTrials.gov, providing valuable insights into the potential and limitations of these innovative therapies.

Implementing these non-antibiotic therapies in resource-constrained settings presents significant cost implications and feasibility challenges. While these therapies offer potential benefits in managing MDR-GI infections, their widespread adoption is limited by factors such as the production and quality control of these treatments, which can be expensive and require technology, infrastructure and expertise often absent in low-resource environments. Moreover, the regulatory landscape for these therapies is still evolving, delaying their introduction and increasing costs. However, local production and sourcing of natural compounds and community-based health initiatives could potentially reduce costs and improve accessibility. Investments in local research and development and international support are crucial to overcoming these barriers and enhancing the feasibility of these alternative therapies in such settings.

Integrating these alternative therapies into existing treatment regimens or antimicrobial stewardship programs constitutes a strategic opportunity to enhance the management of infections and mitigate the current increase in antimicrobial resistance, as it supports the judicious use of antibiotics and provides alternative or complementary options that reduce the use of conventional broad-spectrum antimicrobials. For its effective integration, healthcare systems should establish precise guidelines including these therapies as part of a holistic treatment approach, ensuring they are used based on robust clinical evidence and within the frameworks designed to promote optimal patient outcomes and sustainability in antimicrobial use.

Integrative approaches, which combine probiotics, natural compounds, and bacteriophages, profit from synergistic effects that enhance treatment efficacy and reduce resistance development. On the other hand, personalized medicine further fine-tunes these interventions by adjusting therapies to patients’ individual microbiome profiles and host-specific conditions. Also, innovative delivery systems and large-scale clinical trials are paramount to validate these therapies. Another relevant aspect is establishing robust regulatory frameworks to support non-antibiotic strategies’ development and clinical adoption.

The conclusions presented in this manuscript highlight the importance of collaborative efforts among researchers, clinicians, and policymakers to address the MDR-GI infection challenges. By integrating diverse therapeutic modalities and leveraging advances in biotechnology, it will be possible to pave the way for a new era of precision and sustainability in managing these infections that will limit the global burden of antibiotic resistance and safeguard the efficacy of antimicrobial treatments for future generations.

## References

[B1] Álvarez-Martínez F. J. Barrajón-Catalán E. Micol V. (2020b). Tackling antibiotic resistance with compounds of natural origin: A comprehensive review. Biomedicines 8, 405. doi: 10.3390/biomedicines8100405 33050619 PMC7601869

[B2] Andreani T. Cheng R. Elbadri K. Ferro C. Menezes T. Dos Santos M. R.. (2024). Natural compounds-based nanomedicines for cancer treatment: Future directions and challenges. Drug Delivery Trans. Res. 14, 2845–2916. doi: 10.1007/s13346-023-01318-y PMC11385056 39003425

[B3] Ballini A. Charitos I. A. Cantore S. Topi S. Bottalico L. Santacroce L. (2023). About functional foods: The probiotics and prebiotics state of art. Antibiotics 12, 635. doi: 10.3390/antibiotics12040635 37106999 PMC10135203

[B4] Bao N. Chen F. Dai D. (2020). The regulation of host intestinal microbiota by polyphenols in the development and prevention of chronic kidney disease. Front. Immunol. 10. doi: 10.3389/fimmu.2019.02981 PMC6960133 31969882

[B5] Berkson J. D. Wate C. E. Allen G. B. Schubert A. M. Dunbar K. E. Coryell M. P.. (2024). Phage-specific immunity impairs efficacy of bacteriophage targeting Vancomycin Resistant *Enterococcus* in a murine model. Nat. Commun. 15, 2993. doi: 10.1038/s41467-024-47192-w 38582763 PMC10998888

[B6] Bernatek M. Żukiewicz-Sobczak W. Lachowicz-Wiśniewska S. Piątek J. (2022). Factors determining effective probiotic activity: evaluation of survival and antibacterial activity of selected probiotic products using an “*in vitro*” study. Nutrients 14, 3323. doi: 10.3390/nu14163323 36014829 PMC9413312

[B7] Bhat S. A. Kaur R. Chauhan A. Pal A. (2022). The microbiome and precision oncology: An emerging paradigm in anticancer therapy. Crit. Rev. Microbiol. 48, 770–783. doi: 10.1080/1040841X.2022.2026423 35164642

[B8] Bhatwalkar S. B. Mondal R. Krishna S. B. N. Adam J. K. Govender P. Anupam R. (2021). Antibacterial properties of organosulfur compounds of garlic (*Allium sativum*). Front. Microbiol. 12. doi: 10.3389/fmicb.2021.613077 PMC8362743 34394014

[B9] Biswasroy P. Pradhan D. Sahu D. K. Sahu A. Ghosh G. Rath G. (2021). Recent advances in clinical utility of probiotics in gastrointestinal tract disorders. Curr. Pharm. Biotechnol. 22, 1559–1573. doi: 10.2174/1389201022666211104125455 33121407

[B10] Bleriot I. Pacios O. Blasco L. Fernández-García L. López M. Ortiz-Cartagena C.. (2024). Improving phage therapy by evasion of phage resistance mechanisms. JAC-Antimicrobial Resistance 6, dlae017. doi: 10.1093/jacamr/dlae017 38343627 PMC10854218

[B11] Borges A. de Freitas V. Mateus N. Fernandes I. Oliveira J. (2020). Solid lipid nanoparticles as carriers of natural phenolic compounds. Antioxidants 9, 998. doi: 10.3390/antiox9100998 33076501 PMC7602534

[B12] Borin J. M. Lee J. J. Gerbino K. R. Meyer J. R. (2023). Comparison of bacterial suppression by phage cocktails, dual-receptor generalists, and coevolutionarily trained phages. Evolutionary Appl. 16, 152–162. doi: 10.1111/eva.13518 PMC9850009 36699129

[B13] Bouyahya A. Chamkhi I. Balahbib A. Rebezov M. Shariati M. A. Wilairatana P.. (2022). Mechanisms, anti-quorum-sensing actions, and clinical trials of medicinal plant bioactive compounds against bacteria: A comprehensive review. Molecules 27, 1484. doi: 10.3390/molecules27051484 35268585 PMC8911727

[B14] Bozidis P. Markou E. Gouni A. Gartzonika K. (2024). Does phage therapy need a pan-phage? Pathogens 13, 522. doi: 10.3390/pathogens13060522 38921819 PMC11206709

[B15] Bretaudeau L. Tremblais K. Aubrit F. Meichenin M. Arnaud I. (2020). Good manufacturing practice (GMP) compliance for phage therapy medicinal products. Front. Microbiol. 11. doi: 10.3389/fmicb.2020.01161 PMC7287015 32582101

[B16] Britton R. A. Hoffmann D. E. Khoruts A. (2021). Probiotics and the microbiome: How can we help patients make sense of probiotics? Gastroenterology 160, 614–623. doi: 10.1053/j.gastro.2020.09.062 33307023

[B17] Brives C. Pourraz J. (2020). Phage therapy as a potential solution in the fight against AMR: Obstacles and possible futures. Palgrave Commun. 6, 1–11. doi: 10.1057/s41599-020-0471-8

[B18] Broncano-Lavado A. Santamaría-Corral G. Esteban J. García-Quintanilla M. (2021). Advances in bacteriophage therapy against relevant multidrug-resistant pathogens. Antibiotics 10, 672. doi: 10.3390/antibiotics10060672 34199889 PMC8226639

[B19] Browne A. J. Kashef Hamadani B. H. Kumaran E. A. Rao P. Longbottom J. Harriss E.. (2020). Drug-resistant enteric fever worldwide to 2018: a systematic review and meta-analysis. BMC Med. 18, 1–22. doi: 10.1186/s12916-020-01878-z 31898501 PMC6941399

[B20] Camilleri M. (2021). Human intestinal barrier: Effects of stressors, diet, prebiotics, and probiotics. Clin. Trans. Gastroenterol. 12, e00308. doi: 10.14309/ctg.0000000000000308 PMC7838004 33492118

[B21] Cheng Y. S. Williamson P. R. Zheng W. (2019). Improving therapy of severe infections through drug repurposing of synergistic combinations. Curr. Opin. Pharmacol. 48, 92–98. doi: 10.1016/j.coph.2019.07.010 31454708 PMC6858965

[B22] Colletti A. Pellizzato M. Cicero A. F. (2023). The possible role of probiotic supplementation in inflammation: A narrative review. Microorganisms 11, 2160. doi: 10.3390/microorganisms11092160 37764004 PMC10535592

[B23] Cook M. A. Wright G. D. (2022). The past, present, and future of antibiotics. Sci. Trans. Med. 14, eabo7793. doi: 10.1126/scitranslmed.abo7793 35947678

[B24] Costa P. Pereira C. Romalde J. L. Almeida A. (2024). A Game of Resistance: War Between Bacteria and Phages and How Phage Cocktails can be the Solution. Virology 599, 110209. doi: 10.1016/j.virol.2024.110209 39186863

[B25] Crimi E. Benincasa G. Cirri S. Mutesi R. Faenza M. Napoli C. (2020). Clinical epigenetics and multidrug-resistant bacterial infections: Host remodelling in critical illness. Epigenetics 15, 1021–1034. doi: 10.1080/15592294.2020.1739964 32290755 PMC7518673

[B26] Cunningham M. Azcarate-Peril M. A. Barnard A. Benoit V. Grimaldi R. Guyonnet D.. (2021). Shaping the future of probiotics and prebiotics. Trends Microbiol. 29, 667–685. doi: 10.1016/j.tim.2021.01.003 33551269

[B27] Dahiya D. Nigam P. S. (2023). Antibiotic-therapy-induced gut dysbiosis affecting gut microbiota–brain axis and cognition: Restoration by intake of probiotics and synbiotics. Int. J. Mol. Sci. 24, 3074. doi: 10.3390/ijms24043074 36834485 PMC9959899

[B28] Damyanova T. Dimitrova P. D. Borisova D. Topouzova-Hristova T. Haladjova E. Paunova-Krasteva T. (2024). An overview of biofilm-associated infections and the role of phytochemicals and nanomaterials in their control and prevention. Pharmaceutics 16, 162. doi: 10.3390/pharmaceutics16020162 38399223 PMC10892570

[B29] Dass R. Bhatia M. Rath G. Dhingra A. K. (2025). Recent developments in oral drug delivery of prokinetic agents: nanoparticles and beyond. Curr. Drug Delivery. doi: 10.2174/0115672018296163240910111938 (In Press, not the final “Version of Record”). 39812056

[B30] Deng R. Chen X. Zhao S. Zhang Q. Shi Y. (2024). The effects and mechanisms of natural products on *Helicobacter pylori* eradication. Front. Cell. Infection Microbiol. 14. doi: 10.3389/fcimb.2024.1360852 PMC10933111 38481665

[B31] Deryabin D. Galadzhieva A. Kosyan D. Duskaev G. (2019). Plant-derived inhibitors of AHL-mediated quorum sensing in bacteria: Modes of action. Int. J. Mol. Sci. 20, 5588. doi: 10.3390/ijms20225588 31717364 PMC6888686

[B32] Dey P. (2024). Good girl goes bad: understanding how gut commensals cause disease. Microbial Pathogenesis 190, 106617. doi: 10.1016/j.micpath.2024.106617 38492827

[B33] Dlamini S. B. Gigante A. M. Hooton S. P. Atterbury R. J. (2023). Efficacy of different encapsulation techniques on the viability and stability of diverse phage under simulated gastric conditions. Microorganisms 11, 2389. doi: 10.3390/microorganisms11102389 37894046 PMC10608910

[B34] Dogra S. K. Doré J. Damak S. (2020). Gut microbiota resilience: Definition, link to health, and strategies for intervention. Front. Microbiol. 11. doi: 10.3389/fmicb.2020.572921 PMC7522446 33042082

[B35] Doub J. B. (2021). Risk of bacteriophage therapeutics to transfer genetic material and contain contaminants beyond endotoxins with clinically relevant mitigation strategies. Infection Drug Resistance 14, 5629–5637. doi: 10.2147/IDR.S341265 34992389 PMC8711558

[B36] Dunne M. Prokhorov N. S. Loessner M. J. Leiman P. G. (2021). Reprogramming bacteriophage host range: Design principles and strategies for engineering receptor-binding proteins. Curr. Opin. Biotechnol. 68, 272–281. doi: 10.1016/j.copbio.2020.09.006 33744824 PMC10163921

[B37] Düzgüneş N. Sessevmez M. Yildirim M. (2021). Bacteriophage therapy of bacterial infections: The rediscovered frontier. Pharmaceuticals 14, 34. doi: 10.3390/ph14010034 33466546 PMC7824886

[B38] Elashiry M. M. Bergeron B. E. Tay F. R. (2023). *Enterococcus faecalis* in secondary apical periodontitis: Mechanisms of bacterial survival and disease persistence. Microbial Pathogenesis 183, 106337. doi: 10.1016/j.micpath.2023.106337 37683835

[B39] Elkhalifa M. E. Ashraf M. Ahmed A. Usman A. Hamdoon A. A. Elawad M. A.. (2024). Polyphenols and their nanoformulations as potential antibiofilm agents against multidrug-resistant pathogens. Future Microbiol. 19, 255–279. doi: 10.2217/fmb-2023-0123 38305223

[B40] Emencheta S. C. Olovo C. V. Eze O. C. Kalu C. F. Berebon D. P. Onuigbo E. B.. (2023). The role of bacteriophages in the gut microbiota: implications for human health. Pharmaceutics 15, 2416. doi: 10.3390/pharmaceutics15102416 37896176 PMC10609668

[B41] Fang Q. Yin X. He Y. Feng Y. Zhang L. Luo H.. (2024). Safety and efficacy of phage application in bacterial decolonisation: a systematic review. Lancet Microbe. 5 (5), e489–e499. doi: 10.1016/S2666-5247(24)00002-8 38452780

[B42] Feng W. Liu J. Ao H. Yue S. Peng C. (2020). Targeting gut microbiota for precision medicine: Focusing on the efficacy and toxicity of drugs. Theranostics 10, 11278. doi: 10.7150/thno.47289 33042283 PMC7532689

[B43] Ferry T. Kolenda C. Briot T. Souche A. Lustig S. Josse J.. (2021). Past and future of phage therapy and phage-derived proteins in patients with bone and joint infection. Viruses 13, 2414. doi: 10.3390/v13122414 34960683 PMC8708067

[B44] Ferry T. Kolenda C. Laurent F. Leboucher G. Merabischvilli M. Djebara S.. (2022). Personalized bacteriophage therapy to treat pandrug-resistant spinal *Pseudomonas aeruginosa* infection. Nat. Commun. 13, 4239. doi: 10.1038/s41467-022-31837-9 35869081 PMC9306240

[B45] Fiorani M. Tohumcu E. Del Vecchio L. E. Porcari S. Cammarota G. Gasbarrini A.. (2023). The influence of *Helicobacter pylori* on human gastric and gut microbiota. Antibiotics 12, 765. doi: 10.3390/antibiotics12040765 37107126 PMC10135037

[B46] Fong K. Mu K. Rheault J. G. Levesque R. C. Kitts D. D. Delaquis P.. (2020). Bacteriophage-insensitive mutants of antimicrobial-resistant Salmonella enterica are altered in their tetracycline resistance and virulence in Caco-2 intestinal cells. Int. J. Mol. Sci. 21, 1883. doi: 10.3390/ijms21051883 32164202 PMC7084636

[B47] Frickenstein A. N. Jones M. A. Behkam B. McNally L. R. (2019). Imaging inflammation and infection in the gastrointestinal tract. Int. J. Mol. Sci. 21, 243. doi: 10.3390/ijms21010243 31905812 PMC6981656

[B48] Garcia-Brand A. J. Quezada V. Gonzalez-Melo C. Bolaños-Barbosa A. D. Cruz J. C. Reyes L. H. (2022). Novel developments on stimuli-responsive probiotic encapsulates: From smart hydrogels to nanostructured platforms. Fermentation 8, 117. doi: 10.3390/fermentation8030117

[B49] Garvey M. (2020). Bacteriophages and the one health approach to combat multidrug resistance: Is this the way? Antibiotics 9, 414. doi: 10.3390/antibiotics9070414 32708627 PMC7400126

[B50] Gencay Y. E. Jasinskytė D. Robert C. Semsey S. Martínez V. Petersen A.Ø.. (2024). Engineered phage with antibacterial CRISPR–Cas selectively reduce E. coli burden in mice. coli burden mice. Nat. Biotechnol. 42, 265–274. doi: 10.1038/s41587-023-01759-y 37142704 PMC10869271

[B51] George A. S. Brandl M. T. (2021). Plant bioactive compounds as an intrinsic and sustainable tool to enhance the microbial safety of crops. Microorganisms 9, 2485. doi: 10.3390/microorganisms9122485 34946087 PMC8704493

[B52] Ghosh C. Sarkar P. Issa R. Haldar J. (2019). Alternatives to conventional antibiotics in the era of antimicrobial resistance. Trends Microbiol. 27, 323–338. doi: 10.1016/j.tim.2018.12.010 30683453

[B53] Giovagnoni G. Rossi B. Tugnoli B. Ghiselli F. Bonetti A. Piva A.. (2020). Thymol and carvacrol downregulate the expression of *Salmonella Typhimurium* virulence genes during an *in vitro* infection on Caco-2 cells. Microorganisms 8, 862. doi: 10.3390/microorganisms8060862 32517327 PMC7355688

[B54] Górski A. Borysowski J. Międzybrodzki R. (2020). Phage therapy: towards a successful clinical trial. Antibiotics 9, 827. doi: 10.3390/antibiotics9110827 33227949 PMC7699228

[B55] Gou H. Z. Zhang Y. L. Ren L. F. Li Z. J. Zhang L. (2022). How do intestinal probiotics restore the intestinal barrier? Front. Microbiol. 13. doi: 10.3389/fmicb.2022.929346 PMC9330398 35910620

[B56] Gray D. A. Wenzel M. (2020). Multitarget approaches against multiresistant superbugs. ACS Infect. Dis. 6, 1346–1365. doi: 10.1021/acsinfecdis.0c00098 32156116 PMC7307902

[B57] Grilc N. K. Sova M. Kristl J. (2021). Drug delivery strategies for curcumin and other natural Nrf2 modulators of oxidative stress-related diseases. Pharmaceutics 13, 2137. doi: 10.3390/pharmaceutics13122137 34959418 PMC8708625

[B58] Grudlewska-Buda K. Bauza-Kaszewska J. Wiktorczyk-Kapischke N. Budzyńska A. Gospodarek-Komkowska E. Skowron K. (2023). Antibiotic resistance in selected emerging bacterial foodborne pathogens—An issue of concern? Antibiotics 12, 880. doi: 10.3390/antibiotics12050880 37237783 PMC10215942

[B59] Grumet L. Tromp Y. Stiegelbauer V. (2020). The development of high-quality multispecies probiotic formulations: from bench to market. Nutrients 12, 2453. doi: 10.3390/nu12082453 32824147 PMC7468868

[B60] Gunaratnam S. Millette M. McFarland L. V. DuPont H. L. Lacroix M. (2021). Potential role of probiotics in reducing *Clostridioides difficile* virulence: Interference with quorum sensing systems. Microbial Pathogenesis 153, 104798. doi: 10.1016/j.micpath.2021.104798 33609647

[B61] Guo P. Zhang K. Ma X. He P. (2020). *Clostridium* species as probiotics: potentials and challenges. J. Anim. Sci. Biotechnol. 11, 1–10. doi: 10.1186/s40104-019-0402-1 32099648 PMC7031906

[B62] Gupta A. Ananthakrishnan A. N. (2021). Economic burden and cost-effectiveness of therapies for Clostridiodes difficile infection: a narrative review. Ther. Adv. Gastroenterol. 14, 17562848211018654. doi: 10.1177/17562848211018654 PMC8170348 34104214

[B63] Gupta U. Dey P. (2023). Rise of the guardians: gut microbial maneuvers in bacterial infections. Life Sci. 330, 121993. doi: 10.1016/j.lfs.2023.121993 37536616

[B64] Gutierrez B. Domingo-Calap P. (2020). Phage therapy in gastrointestinal diseases. Microorganisms 8, 1420. doi: 10.3390/microorganisms8091420 32947790 PMC7565598

[B65] Harutyunyan S. Neuhauser I. Mayer A. Aichinger M. Szijártó V. Nagy G.. (2020). Characterization of shigetec, a novel live attenuated combined vaccine against shigellae and etec. Vaccines 8, 689. doi: 10.3390/vaccines8040689 33207794 PMC7712393

[B66] Hasanuzzaman M. Bang C. S. Gong E. J. (2024). Antibiotic resistance of *Helicobacter pylori*: Mechanisms and clinical implications. J. Korean Med. Sci. 39, e44. doi: 10.3346/jkms.2024.39.e44 38288543 PMC10825452

[B67] Hasan M. Ahn J. (2022). Evolutionary dynamics between phages and bacteria as a possible approach for designing effective phage therapies against antibiotic-resistant bacteria. Antibiotics 11 (7), 915. doi: 10.3390/antibiotics11070915 35884169 PMC9311878

[B68] Hassan A. Y. Lin J. T. Ricker N. Anany H. (2021). The age of phage: friend or foe in the new dawn of therapeutic and biocontrol applications? Pharmaceuticals 14 (3), 199. doi: 10.3390/ph14030199 33670836 PMC7997343

[B69] Hatfull G. F. Dedrick R. M. Schooley R. T. (2022). Phage therapy for antibiotic-resistant bacterial infections. Annu. Rev. Med. 73, 197–211. doi: 10.1146/annurev-med-080219-122208 34428079

[B70] Heinrich M. Appendino G. Efferth T. F\u00fcrst R. Izzo A. A. Kayser O.. (2020). Best practice in research\u2014overcoming common challenges in phytopharmacological research. J. Ethnopharmacology 246, 112230. doi: 10.1016/j.jep.2020.112230 31526860

[B71] Heuler J. Fortier L. C. Sun X. (2021). *Clostridioides difficile* phage biology and application. FEMS Microbiol. Rev. 45, fuab012. doi: 10.1093/femsre/fuab012 33580957 PMC8498794

[B72] Hotinger J. A. Morris S. T. May A. E. (2021). The case against antibiotics and for anti-virulence therapeutics. Microorganisms 9, 2049. doi: 10.3390/microorganisms9102049 34683370 PMC8537500

[B73] Hu Y. Lin Q. Zhao H. Li X. Sang S. McClements D. J.. (2023). Bioaccessibility and bioavailability of phytochemicals: Influencing factors, improvements, and evaluations. Food Hydrocolloids 135, 108165. doi: 10.1016/j.foodhyd.2022.108165

[B74] Hu Q. Peng Z. Li L. Zou X. Xu L. Gong J.. (2020). The efficacy of berberine-containing quadruple therapy on *Helicobacter pylori* eradication in China: A systematic review and meta-analysis of randomized clinical trials. Front. Pharmacol. 10. doi: 10.3389/fphar.2019.01694 PMC7010642 32116685

[B75] Hussain Y. Alam W. Ullah H. Dacrema M. Daglia M. Khan H.. (2022). Antimicrobial potential of curcumin: Therapeutic potential and challenges to clinical applications. Antibiotics 11, 322. doi: 10.3390/antibiotics11030322 35326785 PMC8944843

[B76] Jain N. (2020). The need for personalized approaches to microbiome modulation. Front. Public Health 8. doi: 10.3389/fpubh.2020.00144 PMC7200995 32411648

[B77] Jain M. Stitt G. Son L. Enioutina E. Y. (2023). Probiotics and their bioproducts: A promising approach for targeting methicillin-resistant *Staphylococcus aureus* and vancomycin-resistant enterococcus. Microorganisms 11, 2393. doi: 10.3390/microorganisms11102393 37894051 PMC10608974

[B78] Javanshir N. Hosseini G. N. G. Sadeghi M. Esmaeili R. Satarikia F. Ahmadian G.. (2021). Evaluation of the function of probiotics, emphasizing the role of their binding to the intestinal epithelium in the stability and their effects on the immune system. Biol. Procedures Online 23, 1–17. doi: 10.1186/s12575-021-00147-8 PMC8903605 34847891

[B79] Javaudin F. Bémer P. Batard E. Montassier E. (2021). Impact of phage therapy on multidrug-resistant *Escherichia coli* intestinal carriage in a murine model. Microorganisms 9, 2580. doi: 10.3390/microorganisms9122580 34946183 PMC8708983

[B80] João J. Lampreia J. Prazeres D. M. F. Azevedo A. M. (2021). Manufacturing of bacteriophages for therapeutic applications. Biotechnol. Adv. 49, 107758. doi: 10.1016/j.bioteChadv.2021.107758 33895333

[B81] Kachur K. Suntres Z. (2020). The antibacterial properties of phenolic isomers, carvacrol and thymol. Crit. Rev. Food Sci. Nutr. 60, 3042–3053. doi: 10.1080/10408398.2019.1672033 31617738

[B82] Karancsi Z. Kovács D. Palkovicsné Pézsa N. Gálfi P. Jerzsele Á. Farkas O. (2022). The impact of quercetin and its methylated derivatives 3-O-methylquercetin and rhamnazin in lipopolysaccharide-induced inflammation in porcine intestinal cells. Antioxidants 11, 1265. doi: 10.3390/antiox11071265 35883756 PMC9312192

[B83] Keikha M. Karbalaei M. (2021). Probiotics as the live microscopic fighters against *Helicobacter pylori* gastric infections. BMC Gastroenterol. 21, 1–18. doi: 10.1186/s12876-021-01860-w 34670526 PMC8527827

[B84] Khalid A. Lin R. C. Iredell J. R. (2021). A phage therapy guide for clinicians and basic scientists: background and highlighting applications for developing countries. Front. Microbiol. 11. doi: 10.3389/fmicb.2020.599906 PMC7904893 33643225

[B85] Khalil I. Walker R. Porter C. K. Muhib F. Chilengi R. Cravioto A.. (2021). Enterotoxigenic *Escherichia coli* (ETEC) vaccines: Priority activities to enable product development, licensure, and global access. Vaccine 39, 4266–4277. doi: 10.1016/j.vaccine.2021.04.018 33965254 PMC8273896

[B86] Khameneh B. Eskin N. M. Iranshahy M. Fazly Bazzaz B. S. (2021). Phytochemicals: A promising weapon in the arsenal against antibiotic-resistant bacteria. Antibiotics 10, 1044. doi: 10.3390/antibiotics10091044 34572626 PMC8472480

[B87] Khaneghah A. M. Abhari K. Eş I. Soares M. B. Oliveira R. B. Hosseini H.. (2020). Interactions between probiotics and pathogenic microorganisms in hosts and foods: A review. Trends Food Sci. Technol. 95, 205–218. doi: 10.1016/j.tifs.2019.11.019

[B88] Kincses A. Ghazal T. S. A. Hohmann J. (2024). Synergistic effect of phenylpropanoids and flavonoids with antibiotics against Gram-positive and Gram-negative bacterial strains. Pharm. Biol. 62, 659–665. doi: 10.1080/13880209.2024.2389105 39126171 PMC11318484

[B89] Kittler S. Mengden R. Korf I. H. Bierbrodt A. Wittmann J. Plötz M.. (2020). Impact of bacteriophage-supplemented drinking water on the E. coli population in the chicken gut. Pathogens 9, 293. doi: 10.3390/pathogens9040293 32316373 PMC7238078

[B90] Köhler T. Luscher A. Falconnet L. Resch G. McBride R. Mai Q. A.. (2023). Personalized aerosolised bacteriophage treatment of a chronic lung infection due to multidrug-resistant *Pseudomonas aeruginosa* . Nat. Commun. 14, 3629. doi: 10.1038/s41467-023-39370-z 37369702 PMC10300124

[B91] Komala M. G. Ong S. G. Qadri M. U. Elshafie L. M. Pollock C. A. Saad S.. (2023). Investigating the regulatory process, safety, efficacy, and product transparency for nutraceuticals in the USA, Europe, and Australia. Foods 12, 427. doi: 10.3390/foods12020427 36673519 PMC9857896

[B92] Konwar A. N. Hazarika S. N. Bharadwaj P. Thakur D. (2022). Emerging non-traditional approaches to combat antibiotic resistance. Curr. Microbiol. 79, 330. doi: 10.1007/s00284-022-02874-4 36155858 PMC9510247

[B93] Korf I. H. Kittler S. Bierbrodt A. Mengden R. Rohde C. Rohde M.. (2020). *In vitro* evaluation of a phage cocktail controlling infections with *Escherichia coli* . Viruses 12, 1470. doi: 10.3390/v12121470 33352791 PMC7768485

[B94] Kosznik-Kwaśnicka K. Stasiłojć M. Grabowski Ł. Zdrojewska K. Węgrzyn G. Węgrzyn A. (2022). Efficacy and safety of phage therapy against *Salmonella enterica* serovars *Typhimurium* and *Enteritidis* estimated using a battery of *in vitro* tests and the Galleria mellonella animal model. Microbiological Res. 261, 127052. doi: 10.1016/j.micres.2022.127052 35533436

[B95] Kovács D. Palkovicsné Pézsa N. Jerzsele Á. Süth M. Farkas O. (2022). Protective effects of grape seed oligomeric proanthocyanidins in IPEC-J2–*Escherichia coli*/*Salmonella Typhimurium* co-culture. Antibiotics 11, 110. doi: 10.3390/antibiotics11010110 35052987 PMC8773002

[B96] Kovács D. Palkovicsné Pézsa N. Móritz A. V. Jerzsele Á. Farkas O. (2024). Effects of luteolin in an *in vitro* model of porcine intestinal infections. Animals 14, 1952. doi: 10.3390/ani14131952 38998064 PMC11240391

[B97] Kumar M. Sarma D. K. Shubham S. Kumawat M. Verma V. Nina P. B.. (2021). Futuristic non-antibiotic therapies to combat antibiotic resistance: A review. Front. Microbiol. 12. doi: 10.3389/fmicb.2021.609459 PMC7870489 33574807

[B98] Kwiatek M. Parasion S. Nakonieczna A. (2020). Therapeutic bacteriophages as a rescue treatment for drug-resistant infections: An *in vivo* studies overview. J. Appl. Microbiol. 128, 985–1002. doi: 10.1111/jam.14513 31778593

[B99] Lamy-Besnier Q. Chaffringeon L. Lourenço M. Payne R. B. Trinh J. T. Schwartz J. A.. (2021). Prophylactic administration of a bacteriophage cocktail is safe and effective in reducing *Salmonella enterica* serovar *Typhimurium* burden in *vivo* . Microbiol. Spectr. 9, 10–1128. doi: 10.1128/Spectrum.00517-21 PMC8552648 34431719

[B100] Liang D. Wu F. Zhou D. Tan B. Chen T. (2024). Commercial probiotic products in public health: Current status and potential limitations. Crit. Rev. Food Sci. Nutr. 64, 6455–6476. doi: 10.1080/10408398.2023.2169858 36688290

[B101] Liang B. Yuan Y. Peng X. J. Liu X. L. Hu X. K. Xing D. M. (2022). Current and future perspectives for *Helicobacter pylori* treatment and management: From antibiotics to probiotics. Front. Cell. Infection Microbiol. 12. doi: 10.3389/fcimb.2022.1042070 PMC9732553 36506013

[B102] Liu A. Garrett S. Hong W. Zhang J. (2024). *Staphylococcus aureus* infections and human intestinal microbiota. Pathogens 13, 276. doi: 10.3390/pathogens13040276 38668232 PMC11053856

[B103] Liu C. Hong Q. Chang R. Y. K. Kwok P. C. L. Chan H. K. (2022). Phage–antibiotic therapy as a promising strategy to combat multidrug-resistant infections and to enhance antimicrobial efficiency. Antibiotics 11, 570. doi: 10.3390/antibiotics11050570 35625214 PMC9137994

[B104] Liu D. Van Belleghem J. D. de Vries C. R. Burgener E. Chen Q. Manasherob R.. (2021). The safety and toxicity of phage therapy: a review of animal and clinical studies. Viruses 13, 1268. doi: 10.3390/v13071268 34209836 PMC8310247

[B105] Liwinski T. Elinav E. (2020). Harnessing the microbiota for therapeutic purposes. Am. J. Transplant. 20, 1482–1488. doi: 10.1111/ajt.15687 31858698

[B106] Łobocka M. Dąbrowska K. Górski A. (2021). Engineered bacteriophage therapeutics: rationale, challenges and future. BioDrugs 35, 255–280. doi: 10.1007/s40259-021-00480-z 33881767 PMC8084836

[B107] Luong T. Salabarria A. C. Roach D. R. (2020). Phage therapy in the resistance era: Where do we stand and where are we going? Clin. Ther. 42, 1659–1680. doi: 10.1016/j.clinthera.2020.07.014 32883528

[B108] Łusiak-Szelachowska M. Międzybrodzki R. Drulis-Kawa Z. Cater K. Knežević P. Winogradow C.. (2022). Bacteriophages and antibiotic interactions in clinical practice: what we have learned so far. J. Biomed. Sci. 29, 23. doi: 10.1186/s12929-022-00806-1 35354477 PMC8969238

[B109] Ma Y. Fu W. Hong B. Wang X. Jiang S. Wang J. (2023). Antibacterial MccM as the major microcin in against pathogenic enterobacteria Escherichia coli Nissle 1917 against pathogenic enterobacteria. Int. J. Mol. Sci. 24, 11688. doi: 10.3390/ijms241411688 37511446 PMC10380612

[B110] MaChado N. D. Fernández M. A. Díaz D. D. (2019). Recent strategies in resveratrol delivery systems. ChemPlusChem 84, 951–973. doi: 10.1002/cplu.201900174 31943987

[B111] Maillard J. Y. Kampf G. Cooper R. (2021). Antimicrobial stewardship of antiseptics that are pertinent to wounds: The need for a united approach. JAC-Antimicrobial Resistance 3, dlab027. doi: 10.1093/jacamr/dlab027 34223101 PMC8209993

[B112] Majumder M. A. A. Rahman S. Cohall D. Bharatha A. Singh K. Haque M.. (2020). Antimicrobial stewardship: fighting antimicrobial resistance and protecting global public health. Infection Drug Resistance 13, 4713–4738. doi: 10.2147/IDR.S269845 33402841 PMC7778387

[B113] Malczak I. Gajda A. (2023). Interactions of naturally occurring compounds with antimicrobials. J. Pharm. Anal. 13 (12), 1452–1470. doi: 10.1016/j.jpha.2023.04.010 38223447 PMC10785267

[B114] Maldonado C. Cazorla S. I. Lemme Dumit J. M. Vélez E. Perdigón G. (2019). Beneficial effects of probiotic consumption on the immune system. Ann. Nutr. Metab. 74, 115–124. doi: 10.1159/000496426 30673668

[B115] Malik D. J. (2021). Approaches for manufacture, formulation, targeted delivery, and controlled release of phage-based therapeutics. Curr. Opin. Biotechnol. 68, 262–271. doi: 10.1016/j.copbio.2020.09.005 33744823

[B116] Manzari-Tavakoli A. Babajani A. Tavakoli M. M. Safaeinejad F. Jafari A. (2024). Integrating natural compounds and nanoparticle-based drug delivery systems: A novel strategy for enhanced efficacy and selectivity in cancer therapy. Cancer Med. 13, e7010. doi: 10.1002/cam4.7010 38491817 PMC10943377

[B117] Mao X. Wu Y. Ma R. Li L. Wang L. Tan Y.. (2023). Oral phage therapy with microencapsulated phage A221 against *Escherichia coli* infections in weaned piglets. BMC Veterinary Res. 19, 165. doi: 10.1186/s12917-023-03724-y PMC10510151 37730566

[B118] Marongiu L. Burkard M. Lauer U. M. Hoelzle L. E. Venturelli S. (2022). Reassessment of historical clinical trials supports the effectiveness of phage therapy. Clin. Microbiol. Rev. 35, e00062–e00022. doi: 10.1128/cmr.00062-22 36069758 PMC9769689

[B119] Mazziotta C. Tognon M. Martini F. Torreggiani E. Rotondo J. C. (2023). Probiotics mechanism of action on immune cells and beneficial effects on human health. Cells 12, 184. doi: 10.3390/cells12010184 36611977 PMC9818925

[B120] Mehta J. Y. O. T. I. Jandaik S. U. Urmila S. (2016). Evaluation of phytochemicals and synergistic interaction between plant extracts and antibiotics for efflux pump inhibitory activity against Salmonella enterica serovar typhimurium strains. Int. J. Pharm. Pharm. Sci. 8, 217–223. doi: 10.22159/ijpps.2016v8i10.14062

[B121] Merenstein D. Pot B. Leyer G. Ouwehand A. C. Preidis G. A. Elkins C. A.. (2023). Emerging issues in probiotic safety: 2023 perspectives. Gut Microbes 15, 2185034. doi: 10.1080/19490976.2023.2185034 36919522 PMC10026873

[B122] Merker M. Tueffers L. Vallier M. Groth E. E. Sonnenkalb L. Unterweger D.. (2020). Evolutionary approaches to combat antibiotic resistance: opportunities and challenges for precision medicine. Front. Immunol. 11. doi: 10.3389/fimmu.2020.01938 PMC7481325 32983122

[B123] Mikucka A. Deptuła A. Bogiel T. Chmielarczyk A. Nurczyńska E. Gospodarek-Komkowska E. (2022). Bacteraemia caused by probiotic strains of *Lacticaseibacillus rhamnosus* — case studies highlighting the need for careful thought before using microbes for health benefits. Pathogens 11 (9), 977. doi: 10.3390/pathogens11090977 36145409 PMC9504050

[B124] Mohammadi A. Khanbabaei H. Zandi F. Ahmadi A. Haftcheshmeh S. M. Johnston T. P.. (2022). Curcumin: A therapeutic strategy for targeting the *Helicobacter pylori*-related diseases. Microbial Pathogenesis 166, 105552. doi: 10.1016/j.micpath.2022.105552 35469998

[B125] Mohammadi M. Saffari M. Siadat S. D. (2023). Phage therapy of antibiotic-resistant strains of Klebsiella pneumoniae, opportunities and challenges from the past to the future. Folia Microbiologica 68, 357–368. doi: 10.1007/s12223-023-01046-y 37036571

[B126] Mortzfeld B. M. Palmer J. D. Bhattarai S. K. Dupre H. L. Mercado-Lubio R. Silby M. W.. (2022). Microcin MccI47 selectively inhibits enteric bacteria and reduces carbapenem-resistant *Klebsiella pneumoniae* colonization *in vivo* when administered via an engineered live biotherapeutic. Gut Microbes 14, 2127633. doi: 10.1080/19490976.2022.2127633 36175830 PMC9542533

[B127] Mousa W. K. Chehadeh F. Husband S. (2022). Recent advances in understanding the structure and function of the human microbiome. Front. Microbiol. 13. doi: 10.3389/fmicb.2022.825338 PMC8851206 35185849

[B128] Mousa W. K. Mousa S. Ghemrawi R. Obaid D. Sarfraz M. Chehadeh F.. (2023). Probiotics modulate host immune response and interact with the gut microbiota: shaping their composition and mediating antibiotic resistance. Int. J. Mol. Sci. 24, 13783. doi: 10.3390/ijms241813783 37762089 PMC10531388

[B129] Mu A. McDonald D. Jarmusch A. K. Martino C. Brennan C. Bryant M.. (2021). Assessment of the microbiome during bacteriophage therapy in combination with systemic antibiotics to treat a case of staphylococcal device infection. Microbiome 9, 1–8. doi: 10.1186/s40168-021-01155-1 33853672 PMC8048313

[B130] Murphy E. J. Rezoagli E. Major I. Rowan N. J. Laffey J. G. (2020). β-glucan metabolic and immunomodulatory properties and potential for clinical application. J. Fungi 6, 356. doi: 10.3390/jof6040356 PMC7770584 33322069

[B131] Murugaiyan J. Kumar P. A. Rao G. S. Iskandar K. Hawser S. Hays J. P.. (2022). Progress in alternative strategies to combat antimicrobial resistance: Focus on antibiotics. Antibiotics 11, 200. doi: 10.3390/antibiotics11020200 35203804 PMC8868457

[B132] Nagel T. Musila L. Muthoni M. Nikolich M. Nakavuma J. L. Clokie M. R. (2022). Phage banks as potential tools to rapidly and cost-effectively manage antimicrobial resistance in the developing world. Curr. Opin. Virol. 53, 101208. doi: 10.1016/j.coviro.2022.101208 35180534 PMC8846552

[B133] Nale J. Y. Thanki A. M. Rashid S. J. Shan J. Vinner G. K. Dowah A. S.. (2022). Diversity, dynamics, and therapeutic application of Clostridioides difficile bacteriophages. Viruses 14, 2772. doi: 10.3390/v14122772 36560776 PMC9784644

[B134] Newman A. M. Arshad M. (2020). The role of probiotics, prebiotics, and synbiotics in combating multidrug-resistant organisms. Clin. Ther. 42, 1637–1648. doi: 10.1016/j.clinthera.2020.07.011 32800382 PMC7904027

[B135] Oliveira M. Antunes W. Mota S. Madureira-Carvalho Á. Dinis-Oliveira R. J. da Silva D. D. (2024). An overview of the recent advances in antimicrobial resistance. Microorganisms 12, 1920. doi: 10.3390/microorganisms12091920 39338594 PMC11434382

[B136] Osei-Owusu H. Rondevaldova J. Houdkova M. Kudera T. Needham T. Mascellani A.. (2024). Evaluation of *in vitro* synergistic effects of tetracycline with alkaloid-related compounds against diarrhoeic bacteria. Int. J. Mol. Sci. 25, 6038. doi: 10.3390/ijms25116038 38892226 PMC11173066

[B137] Pagnossa J. P. Rocchetti G. de Abreu Martins H. H. Bezerra J. D. P. Batiha G. E. S. El-Masry E. A.. (2021). Morphological and metabolomics impact of sublethal doses of natural compounds and its nanoemulsions in *Bacillus cereus* . Food Res. Int. 149, 110658. doi: 10.1016/j.foodres.2021.110658 34600660

[B138] Pal R. Athamneh A. I. Deshpande R. Ramirez J. A. Adu K. T. Muthuirulan P.. (2023). Probiotics: Insights and new opportunities for *Clostridioides difficile* intervention. Crit. Rev. Microbiol. 49, 414–434. doi: 10.1080/1040841X.2022.2163577 35574602 PMC9743071

[B139] Panda S. K. Buroni S. Swain S. S. Bonacorsi A. da Fonseca Amorim E. A. Kulshrestha M.. (2022). Recent advances to combat ESKAPE pathogens with special reference to essential oils. Front. Microbiol. 13. doi: 10.3389/fmicb.2022.1029098 PMC9763703 36560948

[B140] Patel P. Garala K. Singh S. Prajapati B. G. Chittasupho C. (2024). Lipid-based nanoparticles in delivering bioactive compounds for improving therapeutic efficacy. Pharmaceuticals 17, 329. doi: 10.3390/ph17030329 38543115 PMC10975431

[B141] Paterson D. L. (2024). Antibacterial agents active against Gram negative bacilli in phase I, II, or III clinical trials. Expert Opin. Investigational Drugs 33, 371–387. doi: 10.1080/13543784.2024.2326028 38445383

[B142] Pathak D. Mazumder A. (2024). Potential of flavonoids as promising phytotherapeutic agents to combat multidrug-resistant infections. Curr. Pharm. Biotechnol. 25 (13), 1664–1692. doi: 10.2174/1389201025666220913105632 38031767

[B143] Pell L. G. Loutet M. G. Roth D. E. Sherman P. M. (2019). Arguments against routine administration of probiotics for NEC prevention. Curr. Opin. Pediatr. 31, 195–201. doi: 10.1097/MOP.0000000000000752 30624281

[B144] Peters V. B. M. Van De Steeg E. Van Bilsen J. Meijerink M. (2019). Mechanisms and immunomodulatory properties of pre-and probiotics. Beneficial Microbes 10, 225–236. doi: 10.3920/BM2018.0133 30827150

[B145] Petrovic Fabijan A. Iredell J. Danis-Wlodarczyk K. Kebriaei R. Abedon S. T. (2023). Translating phage therapy into the clinic: Recent accomplishments but continuing challenges. PloS Biol. 21, e3002119. doi: 10.1371/journal.pbio.3002119 37220114 PMC10204993

[B146] Piazza S. Martinelli G. Fumagalli M. Pozzoli C. Maranta N. Giavarini F.. (2023). Ellagitannins from Castanea sativa Mill. Leaf Extracts Impair *Helicobacter pylori* Viability and Infection-Induced Inflammation in Human Gastric Epithelial Cells. Nutrients 15, 1504. doi: 10.3390/nu15061504 36986236 PMC10056456

[B147] Piel D. Bruto M. Labreuche Y. Blanquart F. Goudenège D. Barcia-Cruz R.. (2022). Phage–host coevolution in natural populations. Nat. Microbiol. 7, 1075–1086. doi: 10.1038/s41564-022-01128-4 35760840

[B148] Pinto R. M. Soares F. A. Reis S. Nunes C. Van Dijck P. (2020). Innovative strategies toward the disassembly of the EPS matrix in bacterial biofilms. Front. Microbiol. 11. doi: 10.3389/fmicb.2020.00952 PMC7264105 32528433

[B149] Plaza-Diaz J. Ruiz-Ojeda F. J. Gil-Campos M. Gil A. (2019). Mechanisms of action of probiotics. Adv. Nutr. 10, S49–S66. doi: 10.1093/advances/nmz049 30721959 PMC6363529

[B150] Qassadi F. I. Zhu Z. Monaghan T. M. (2023). Plant-derived products with therapeutic potential against gastrointestinal bacteria. Pathogens 12, 333. doi: 10.3390/pathogens12020333 36839605 PMC9967904

[B151] Rahman M. N. Barua N. Tin M. C. Dharmaratne P. Wong S. H. Ip M. (2024). The use of probiotics and prebiotics in decolonizing pathogenic bacteria from the gut; a systematic review and meta-analysis of clinical outcomes. Gut Microbes 16, 2356279. doi: 10.1080/19490976.2024.2356279 38778521 PMC11123511

[B152] Rathinasabapathy T. Lomax J. Srikanth K. Esposito D. Kay C. D. Komarnytsky S. (2022). Effect of wild blueberry metabolites on biomarkers of gastrointestinal and immune health in *vitro* . Immuno 2, 293–306. doi: 10.3390/immuno2020022

[B153] Rose E. C. Odle J. Blikslager A. T. Ziegler A. L. (2021). Probiotics, prebiotics and epithelial tight junctions: A promising approach to modulate intestinal barrier function. Int. J. Mol. Sci. 22, 6729. doi: 10.3390/ijms22136729 34201613 PMC8268081

[B154] Rosellini E. Cascone M. G. (2023). Microfluidic fabrication of natural polymer-based scaffolds for tissue engineering applications: A review. Biomimetics 8, 74. doi: 10.3390/biomimetics8010074 36810405 PMC9944883

[B155] Rueda-Robles A. Rodríguez-Lara A. Meyers M. S. Sáez-Lara M. J. Álvarez-Mercado A. I. (2022). Effect of probiotics on host-microbiota in bacterial infections. Pathogens 11, 986. doi: 10.3390/pathogens11090986 36145418 PMC9500725

[B156] Sakarikou C. Kostoglou D. Simões M. Giaouris E. (2020). Exploitation of plant extracts and phytochemicals against resistant Salmonella spp. in biofilms. Food Res. Int. 128, 108806. doi: 10.1016/j.foodres.2019.108806 31955766

[B157] Salam M. A. Al-Amin M. Y. Salam M. T. Pawar J. S. Akhter N. Rabaan A. A.. (2023). Antimicrobial resistance: A growing serious threat for global public health. Healthcare 11, 1946. doi: 10.3390/healthcare11131946 37444780 PMC10340576

[B158] Salazar K. C. Ma L. Green S. I. Zulk J. J. Trautner B. W. Ramig R. F.. (2021). Antiviral resistance and phage counter-adaptation to antibiotic-resistant extraintestinal pathogenic *Escherichia coli* . mBio 12, 10–1128. doi: 10.1128/mBio.00219-21 PMC8092219 33906920

[B159] Salih S. Elliyanti A. Alkatheeri A. AlYafei F. Almarri B. Khan H. (2023). The role of molecular imaging in personalized medicine. J. Personalized Med. 13, 369. doi: 10.1016/j.ebiom.2015.12.023 PMC9959741 36836603

[B160] Sarker S. A. Sultana S. Reuteler G. Moine D. Descombes P. Charton F.. (2016). Oral phage therapy of acute bacterial diarrhea with two coliphage preparations: a randomized trial in children from bangladesh. EBioMedicine 4, 124–137. doi: 10.1016/j.ebiom.2015.12.023 26981577 PMC4776075

[B161] Sassone-Corsi M. Nuccio S. P. Liu H. Hernandez D. Vu C. T. Takahashi A. A.. (2016). Microcins mediate competition among Enterobacteriaceae in the inflamed gut. Nature 540, 280–283. doi: 10.1038/nature20557 27798599 PMC5145735

[B162] Sathianarayanan S. Ammanath A. V. Biswas R. Anita B. Sukumaran S. Venkidasamy B. (2022). A new approach against *Helicobacter pylori* using plants and its constituents: A review study. Microbial Pathogenesis 168, 105594. doi: 10.1016/j.micpath.2022.105594 35605740

[B163] SChinas G. Skintzi K. De Lastic A. L. Rodi M. Gogos C. Mouzaki A.. (2023). Patterns, cost, and immunological response of MDR vs non-MDR bacteremia: A prospective cohort study. Pathog. 12, 1044. doi: 10.3390/pathogens12081044 PMC10458260 37624004

[B164] Scotti F. Booker A. Heinrich M. (2019). St. John\u2019s Wort (*Hypericum perforatum*) products\u2014How variable is the primary material? Front. Plant Sci. 9. doi: 10.3389/fpls.2018.01973 PMC6357942 30740121

[B165] Seo H. Duan Q. Zhang W. (2020). Vaccines against gastroenteritis, current progress and challenges. Gut Microbes 11, 1486–1517. doi: 10.1080/19490976.2020.1770666@kgmi20.2020.12.issue-SI1 32552414 PMC7524147

[B166] Silva D. R. Sardi J. D. C. O. de Souza Pitangui N. Roque S. M. da Silva A. C. B. Rosalen P. L. (2020). Probiotics as an alternative antimicrobial therapy: Current reality and future directions. J. Funct. Foods 73, 104080. doi: 10.1016/j.jff.2020.104080

[B167] Singh A. Padmesh S. Dwivedi M. Kostova I. (2022). How good are bacteriophages as an alternative therapy to mitigate biofilms of nosocomial infections? Infection Drug Resistance 15, 503–532. doi: 10.2147/IDR.S328646 35210792 PMC8860455

[B168] Song M. Liu Y. Li T. Liu X. Hao Z. Ding S.. (2021). Plant natural flavonoids against multidrug-resistant pathogens. Advanced Sci. 8, 2100749. doi: 10.1002/advs.202100749 PMC8336499 34041861

[B169] Stavropoulou E. Bezirtzoglou E. (2020). Probiotics in medicine: A long debate. Front. Immunol. 11. doi: 10.3389/fimmu.2020.02192 PMC7544950 33072084

[B170] Suh G. A. Lodise T. P. Tamma P. D. Knisely J. M. Alexander J. Aslam S. (2022). Considerations for the use of phage therapy in clinical practice. Antimicrobial Agents Chemotherapy 66, e02071–e02021. doi: 10.1128/aac.02071-21 35041506 PMC8923208

[B171] Suleman M. Clark J. R. Bull S. Jones J. D. (2024). Ethical argument for establishing good manufacturing practice for phage therapy in the UK. J. Med. Ethics, 1–5. doi: 10.1136/jme-2023-109423 38342498 PMC12171505

[B172] Talapko J. Škrlec I. (2020). The principles, mechanisms, and benefits of unconventional agents in the treatment of biofilm infection. Pharmaceuticals 13, 299. doi: 10.3390/ph13100299 33050521 PMC7600518

[B173] Tan S. Yan F. Li Q. Liang Y. Yu J. Li Z.. (2020). Chlorogenic acid promotes autophagy and alleviates *Salmonella Typhimurium* infection through the lncRNA GAS5/miR-23a/PTEN axis and the p38 MAPK pathway. Front. Cell Dev. Biol. 8. doi: 10.3389/fcell.2020.552020 PMC7682651 33240872

[B174] Tang C. Lu Z. (2019). Health-promoting activities of probiotics. J. Food Biochem. 43, e12944. doi: 10.1111/jfbc.12944 31368544

[B175] Tasaka M. Saito K. Matuo Y. (2023). Pro-, pre-, and post-biotics related to gut microbiota and immune system. Japanese J. Food Chem. Saf. 30, 43.

[B176] Tavares T. D. Antunes J. C. Padrão J. Ribeiro A. I. Zille A. Amorim M. T. P.. (2020). Activity of specialized biomolecules against gram-positive and gram-negative bacteria. Antibiotics 9, 314. doi: 10.3390/antibiotics9060314 32526972 PMC7344598

[B177] Teixeira P. V. Fernandes E. Soares T. B. Adega F. Lopes C. M. Lúcio M. (2023). Natural compounds: Co-delivery strategies with chemotherapeutic agents or nucleic acids using lipid-based nanocarriers. Pharmaceutics 15, 1317. doi: 10.3390/pharmaceutics15041317 37111802 PMC10141470

[B178] Terwilliger A. Clark J. Karris M. Hernandez-Santos H. Green S. Aslam S.. (2021). Phage therapy related microbial succession associated with successful clinical outcome for a recurrent urinary tract infection. Viruses 13, 2049. doi: 10.3390/v13102049 34696479 PMC8541385

[B179] Thanki A. M. Mignard G. Atterbury R. J. Barrow P. Millard A. D. Clokie M. R. (2022). Prophylactic delivery of a bacteriophage cocktail in feed significantly reduces *Salmonella* colonization in pigs. Microbiol. Spectr. 10, e00422–e00422. doi: 10.1128/spectrum.00422-22 35579475 PMC9241700

[B180] Todd K. (2019). The promising viral threat to bacterial resistance: The uncertain patentability of phage therapeutics and the necessity of alternative incentives. Duke Law J. 68, 767–805. doi: 10.3316/agispt.20240102101366 30649837

[B181] Topka-Bielecka G. Dydecka A. Necel A. Bloch S. Nejman-Faleńczyk B. Węgrzyn G.. (2021). Bacteriophage-derived depolymerases against bacterial biofilm. Antibiotics 10, 175. doi: 10.3390/antibiotics10020175 33578658 PMC7916357

[B182] Trifan A. Luca S. V. Greige-Gerges H. Miron A. Gille E. Aprotosoaie A. C. (2020). Recent advances in tackling microbial multidrug resistance with essential oils: Combinatorial and nano-based strategies. Crit. Rev. Microbiol. 46, 338–357. doi: 10.1080/1040841X.2020.1720080 32608293

[B183] Underwood M. A. (2019). Probiotics and the prevention of necrotizing enterocolitis. J. Pediatr. Surg. 54, 405–412. doi: 10.1016/j.jpedsurg.2018.08.062 30241961

[B184] Vaitekenas A. Tai A. S. Ramsay J. P. Stick S. M. Kicic A. (2021). Pseudomonas aeruginosa resistance to bacteriophages and its prevention by strategic therapeutic cocktail formulation. Antibiotics 10, 145. doi: 10.3390/antibiotics10020145 33540528 PMC7912912

[B185] Vanegas-Múnera J. M. Jiménez-Quiceno J. N. (2020). Resistencia antimicrobiana en el siglo XXI: ¿Hacia una era postantibiótica? Revista Facultad Nacional de Salud Publica 38 (1), e337759. doi: 10.17533/udea.rfnsp.v38n1e337759

[B186] Van Zyl W. F. Deane S. M. Dicks L. M. (2020). Molecular insights into probiotic mechanisms of action employed against intestinal pathogenic bacteria. Gut Microbes 12, 1831339. doi: 10.1080/19490976.2020.1831339 33112695 PMC7595611

[B187] Vaou N. Stavropoulou E. Voidarou C. Tsigalou C. Bezirtzoglou E. (2021). Towards advances in medicinal plant antimicrobial activity: A review study on challenges and future perspectives. Microorganisms 9, 2041. doi: 10.3390/microorganisms9102041 34683362 PMC8541629

[B188] Verbeken G. Pirnay J. P. (2022). European regulatory aspects of phage therapy: Magistral phage preparations. Curr. Opin. Virol. 52, 24–29. doi: 10.1016/j.coviro.2022.02.005 34801778

[B189] Verediano T. A. Stampini Duarte Martino H. Dias Paes M. C. Tako E. (2021). Effects of anthocyanin on intestinal health: A systematic review. Nutrients 13, 1331. doi: 10.3390/nu13041331 33920564 PMC8074038

[B190] Vilas-Boas A. A. Pintado M. Oliveira A. L. (2021). Natural bioactive compounds from food waste: Toxicity and safety concerns. Foods 10, 1564. doi: 10.3390/foods10071564 34359434 PMC8304211

[B191] Walker R. Kaminski R. W. Porter C. Choy R. K. White J. A. Fleckenstein J. M.. (2021). Vaccines for protecting infants from bacterial causes of diarrheal disease. Microorganisms 9, 1382. doi: 10.3390/microorganisms9071382 34202102 PMC8303436

[B192] Walter N. Mirzaei M. Deng L. Willy C. Alt V. Rupp M. (2024). The potential of bacteriophage therapy as an alternative treatment approach for antibiotic-resistant infections. Med. Principles Pract. 33, 1–9. doi: 10.1159/000534717 PMC10896615 37879316

[B193] Watkins R. R. (2022). Antibiotic stewardship in the era of precision medicine. JAC-Antimicrobial Resistance 4, dlac066. doi: 10.1093/jacamr/dlac066 35733911 PMC9209748

[B194] Weersma R. K. Zhernakova A. Fu J. (2020). Interaction between drugs and the gut microbiome. Gut 69, 1510–1519. doi: 10.1136/gutjnl-2019-320204 32409589 PMC7398478

[B195] Xiao G. Li J. Sun Z. (2023). The combination of antibiotic and non-antibiotic compounds improves antibiotic efficacy against multidrug-resistant bacteria. Int. J. Mol. Sci. 24, 15493. doi: 10.3390/ijms242015493 37895172 PMC10607837

[B196] Yang Q. Le S. Zhu T. Wu N. (2023). Regulations of phage therapy across the world. Front. Microbiol. 14. doi: 10.3389/fmicb.2023.1250848 PMC10588630 37869667

[B197] Yang M. Meng F. Gu W. Li F. Tao Y. Zhang Z.. (2020). Effects of natural products on bacterial communication and network-quorum sensing. BioMed. Res. Int. 2020, 8638103. doi: 10.1155/2020/8638103 32596389 PMC7273434

[B198] Yao M. Xie J. Du H. McClements D. J. Xiao H. Li L. (2020). Progress in microencapsulation of probiotics: A review. Compr. Rev. Food Sci. Food Saf. 19, 857–874. doi: 10.1111/1541-4337.12535 33325164

[B199] Zafer M. M. Gamal A. M. Sabrin R. M. I. Soumya G. Charné B. Mahmoud A. E. (2024). Biofilm-mediated infections by multidrug-resistant microbes: A comprehensive exploration and forward perspectives. Arch. Microbiol. 206, 101. doi: 10.1007/s00203-023-03826-z 38353831 PMC10867068

[B200] Zawistowska-Rojek A. Zaręba T. Tyski S. (2022). Microbiological testing of probiotic preparations. Int. J. Environ. Res. Public Health 19, 5701. doi: 10.3390/ijerph19095701 35565098 PMC9099753

[B201] Zhang X. Guo J. Cheng F. Li S. (2021). Cytochrome P450 enzymes in fungal natural product biosynthesis. Natural Product Rep. 38, 1072–1099. doi: 10.1039/D0NP00098E 33710221

[B202] Zhang W. Lian Y. Li Q. Sun L. Chen R. Lai X.. (2020). Preventative and therapeutic potential of flavonoids in peptic ulcers. Molecules 25, 4626. doi: 10.3390/molecules25204626 33050668 PMC7594042

[B203] Zhou H. Wang W. Cai L. Yang T. (2023). Potentiation and mechanism of berberine as an antibiotic adjuvant against multidrug-resistant bacteria. Infection Drug Resistance 16, 7313–7326. doi: 10.2147/IDR.S426805 38023403 PMC10676105

