# Supplementary material for: Non-antibiotic therapies for multidrug-resistant gastrointestinal infections: an overview of the use of probiotics, natural compounds, and bacteriophages
Source: Front Antibiot. 2025 May 6;4:1554061. doi: 10.3389/frabi.2025.1554061 (PMC12089134; doi:10.3389/frabi.2025.1554061)
Supplement: Supplementary file 1 [file Table1.docx]

**Supplementary Table 1.** Antibiotic Resistance Patterns and Mechanisms in Major Multidrug-Resistant Gastrointestinal (MDR-GI) Pathogens

| **Bacteria** | **Antibiotic Classes with resistance** | **Resistance acquisition** | **Mechanism of Resistance** | **References** |
| --- | --- | --- | --- | --- |
| *Clostridioides difficile* | - Fluoroquinolones (e.g., ciprofloxacin, levofloxacin, moxifloxacin) - Glycopeptide antibiotics (e.g., vancomycin - low) - Lincomycin antibiotics (e.g, clindamycin) - Macrolides (e.g., erythromycin, clarithromycin – low, fidaxomicin – very low) - Metronidazole (low) - Tetracyclines (e.g., doxycycline – low) | - Horizontally acquired genes - Point mutations | - Drug alteration - Extrusion of drugs via efflux pumps - Modification of the target site | Teng et al., 2023  Dureja et al., 2022  Saha et al., 2019 |
| *Helicobacter pylori* | - Beta-lactams (e.g., amoxicillin – very low) - Fluoroquinolones (e.g., levofloxacin) - Macrolides (e.g., clarithromycin) - Metronidazole (e.g., nitroimidazole - low) - Tetracyclines (low) | - Horizontally acquired genes - Point mutations - Natural transformation | - Biofilm formation - Extrusion of drugs via efflux pumps - Gene mutations (nucleic acid synthesis‐related gene, rRNA coding gene, cell wall synthesis‐related gene) | Huang et al., 2023  Lin et al., 2023  Mehrotra et al., 2021  Kasahun et al., 2020  Smith et al., 2019 |
| *Listeria monocytogenes* | - Aminoglycosides (e.g., gentamicin) - β-lactams (e.g., amoxicillin, ampicillin, oxacillin) - Chloramphenicol - Lincomycin antibiotics (e.g, clindamycin) - Macrolides (e.g., erythromycin) - Phosphonic antibiotics (e.g., fosfomycin) - Sulfonamides and Trimethoprim (e.g., trimethoprim, Trimethoprim-sulfamethoxazole) - Tetracyclines (e.g., tetracycline) | - Horizontally acquired genes (plasmids, transposons) | - Drug alteration (enzymatic hydrolysis) - Modification of the target site - Extrusion of drugs via efflux pumps - Reduced cell membrane permeability | Rippa et al, 2024  Ge et al., 2022  Rostamian et al., 2022  Andriyanov et al., 2021  Keet et al., 2021  Ulusoy et al., 2019 |
| *Camplylobacter* spp.  (mainly *C. jejuni* and *C. coli*) | - Aminoglycosides - β-lactams (e.g., ampicillin) - Chloramphenicols - Fluoroquinolones (e.g., ciprofloxacin, gatifloxacin) - Lincomycin antibiotics (e.g, clindamycin) - Macrolides (e.g., azithromycin, clarithromycin, erythromycin,) - Tetracyclines (e.g., doxycycline, tetracycline) | - Horizontally acquired genes | - Drug alteration (enzymatic hydrolysis) - Gene mutations (DNA gyrase, rRNA coding gene) - Modification of the target site - Reduced cell membrane permeability | Chibwe et al., 2023  Qin et al., 2023  Aleksić et al., 2021  Rivera-Mendoza et al., 2020 |
| *Salmonella s*pp. | - Aminoglycosides (e.g., gentamicin – low, streptomycin) - β-lactams (e.g., ampicillin, penicillins, cephalosporins) - Fluoroquinolones (e.g., ciprofloxacin, nalidixic acid) - Macrolides (e.g., azithromycin – emerging) - Sulfonamides - Tetracyclines (e.g., tetracycline) - Chloramphenicol | - Horizontal gene transfer - Lysogenic phages - Point mutations | - Production of extended-spectrum β-lactamases (ESBLs) - Gene mutations (DNA gyrase) - Extrusion of drugs via efflux pumps | Wójcicki et al., 2021  Wang et al., 2019 |
| *Shigella spp.*  (mainly *S. flexneri* and *S. sonnei*) | - β-lactams (e.g., ampicillin) - Chloramphenicol - Fluoroquinolones (e.g., nalidixic acid) - Macrolides (e.g., azithromycin) - Sulfonamides and Trimethoprim (e.g., trimethoprim-sulfamethoxazole) - Tetracyclines | - Horizontally acquired genes (plasmids) | - Drug alteration (enzymatic hydrolysis) - Extrusion of drugs via efflux pumps - Gene mutations (DNA topoisomerase IV) - Modification of the target site - Reduced cell membrane permeability | Bose et al., 2024  Ranjbar et al., 2019 |
| *Escherichia coli*  (e.g., LCT-EC001 and ST131) | - Aminoglycosides (e.g., gentamicin, amikacin, streptomycin) - β-lactams (e.g., amoxicillin, ampicillin, ampicillin-sulbactam, cephalosporins) - Cephalosporin (e.g., cefotaxime, cefatriaxone, cefuroxime) - Chloramphenicol - Fluoroquinolones (e.g., ciprofloxacin, levofloxacin, nalidixic acid) - Sulfonamides and Trimethoprim (e.g., trimethoprim, Trimethoprim-sulfamethoxazole) - Carbapenems - Tetracyclines (e.g., oxytetracycline, tetracycline) | - Horizontally acquired genes (plasmids, transposons) | - Production of extended-spectrum β-lactamases (ESBLs) and carbapenemases - Gene mutations (DNA gyrase/topoisomerase IV, porins) - Modifying enzymes - Extrusion of drugs via efflux pumps - Ribosomal protection - Activation of stress response systems, to withstand antibiotic pressure - Capsule and biofilm production | Asrollahian et al., 2024  Johnston et al., 2021  Nji et al., 2021  Beattie et al., 2020  Stephens et al., 2020  Pouwels et al., 2019 |

**References**

Aleksić, E., Miljković-Selimović, B., Tambur, Z., Aleksić, N., Biočanin, V., and Avramov, S. (2021). Resistance to antibiotics in thermophilic *Campylobacters*. Front. Med., 8, 763434. doi:10.3389/fmed.2021.763434
Andriyanov, P. A., Zhurilov, P. A., Liskova, E. A., Karpova, T. I., Sokolova, E. V., Yushina, Y. K., et al. (2021). Antimicrobial resistance of *Listeria monocytogenes* strains isolated from humans, animals, and food products in Russia in 1950–1980, 2000–2005, and 2018–2021. Antibiotics, 10, 1206. doi: 10.3390/antibiotics10101206
Asrollahian, S., Graham, J. P., and Halaji, M. (2024). A review of the mechanisms that confer antibiotic resistance in pathotypes of *E. coli*. Front. Cell. Infect. Microbiol., 14, 1387497. doi: 10.3389/fcimb.2023.1387497
Beattie, R. E., Bakke, E., Konopek, N., Thill, R., Munson, E., and Hristova, K. R. (2020). Antimicrobial resistance traits of *Escherichia coli* isolated from dairy manure and freshwater ecosystems are similar to one another but differ from associated clinical isolates. Microorganisms, 8, 747. doi: 10.3390/microorganisms8050747
Bose, P., Chowdhury, G., Halder, G., Ghosh, D., Deb, A. K., Kitahara, K., et al. (2024). Prevalence and changing antimicrobial resistance profiles of *Shigella spp.* isolated from diarrheal patients in Kolkata during 2011–2019. PLOS Neglected Tropical Diseases, 18, e0011964. doi: 10.1371/journal.pntd.0011964
Chibwe, M., Odume, O. N., & Nnadozie, C. F. (2023). Assessment of risk of exposure to Campylobacter species and their antibiotic-resistant genes from selected rivers in the eastern cape, South Africa. Environ. Poll.,*338*, 122625. doi: 10.1016/j.envpol.2023.122625
Dureja, C., Olaitan, A. O., and Hurdle, J. G. (2022). Mechanisms and impact of antimicrobial resistance in *Clostridioides difficile*. Curr. Opin. Microbiol., 66, 63–72. doi: 10.1016/j.mib.2022.01.007
Ge, H., Wang, Y., and Zhao, X. (2022). Research on the drug resistance mechanism of foodborne pathogens. Microbial Pathogenesis, 162, 105306. doi: 10.1016/j.micpath.2022.105306
Huang, Z., Zhu, Y., Li, X., Yao, Z., and Ge, R. (2023). The mechanisms of metronidazole resistance of *Helicobacter pylori*: A transcriptomic and biochemical study. Microb. Pathog., 183, 106303. doi: 10.1016/j.micpath.2023.106303
Johnston, B. D., Thuras, P., Porter, S. B., Anacker, M., VonBank, B., Vagnone, P. S., et al. (2021). Global molecular epidemiology of carbapenem-resistant *Escherichia coli*. *Eur. J.*Clin. Microbiol. Infect. Dis*.*, 1–13. doi: 10.1007/s10096-021-04217-2
Kasahun, G. G., Demoz, G. T., and Desta, D. M. (2020). Primary resistance pattern of *Helicobacter pylori* to antibiotics in the adult population: A systematic review. Infect. Drug Resist. 13, 1567–1573. doi: 10.2147/IDR.S257613
Keet, R., and Rip, D. (2021). *Listeria monocytogenes* isolates from Western Cape, South Africa exhibit resistance to multiple antibiotics and contradict certain global resistance patterns. AIMS Microbiol*.* 7, 40–53. doi: 10.3934/microbiol.2021003
Lin, Y., Ni, Y., Shen, Y., Zheng, J., Zhang, L., and Zhang, Y. (2023). Antimicrobial resistance and genotypic characteristics of *Helicobacter pylori* clinical isolates in China: A multicenter study. Helicobacter, 28, e12976. doi: 10.1111/hel.12976
Mehrotra, T., Devi, T. B., Kumar, S., Talukdar, D., Karmakar, S. P., Kothidar, A., et al. (2021). Antimicrobial resistance and virulence in *Helicobacter pylori*: Genomic insights. Genomics, 113, 3951–3966. doi: 10.1016/j.ygeno.2021.09.011
Nji, E., Kazibwe, J., Hambridge, T., Joko, C. A., Larbi, A. A., Damptey, L. A. O., et al. (2021). High prevalence of antibiotic resistance in commensal *Escherichia coli* from healthy children in rural Uganda: implications for community health. BMC Microbiol., 21, 1–13. doi: 10.1186/s12866-021-02279-4
Pouwels, K. B., Muller-Pebody, B., Smieszek, T., Hopkins, S., and Robotham, J. V. (2019). Selection and co-selection of antibiotic resistances among *Escherichia coli* by antibiotic use in primary care: An ecological analysis. PloS One ,14, e0218134. doi: 10.1371/journal.pone.0218134
Qin, X., Wang, X., and Shen, Z. (2023). The rise of antibiotic resistance in *Campylobacter*. Curr. Opin. Gastroenterol., 39, 9–15. doi: 10.1097/MOG.0000000000000888
Ranjbar, R., and Farahani, A. (2019). *Shigella*: Antibiotic-resistance mechanisms and new horizons for treatment. Infect. Drug Resist., 12, 3137–3167. doi: 10.2147/IDR.S219755
Rippa, A., Bilei, S., Peruzy, M. F., Marrocco, M. G., Leggeri, P., Bossù, T., et al. (2024). Antimicrobial resistance of *Listeria monocytogenes* strains isolated in food and food-processing environments in Italy. Antibiotics, 13, 525. doi: 10.3390/antibiotics13060525
Rivera-Mendoza, D., Martínez-Flores, I., Santamaría, R. I., Lozano, L., Bustamante, V. H., and Pérez-Morales, D. (2020). Genomic analysis reveals the genetic determinants associated with antibiotic resistance in the zoonotic pathogen *Campylobacter* spp. distributed globally. Front. Microbiol., 11, 513070. doi: 10.3389/fmicb.2020.513070
Rostamian, M., Kooti, S., Mohammadi, B., Salimi, Y., and Akya, A. (2022). A systematic review and meta-analysis of *Listeria monocytogenes* isolated from human and non-human sources: The antibiotic susceptibility aspect. Diagn. Microbiol. Infect. Dis., 102, 115634. doi: 10.1016/j.diagmicrobio.2021.115634
Saha, S., Kapoor, S., Tariq, R., Schuetz, A. N., Tosh, P. K., Pardi, D. S., et al. (2019). Increasing antibiotic resistance in *Clostridioides difficile*: A systematic review and meta-analysis. Anaerobe, 58, 35–46. doi: 10.1016/j.anaerobe.2019.02.005
Smith, S. M., O’Morain, C., and McNamara, D. (2019). *Helicobacter pylori* resistance to current therapies. Curr. Opin. Gastroenterol., 35, 6–13. doi: 10.1097/MOG.0000000000000505
Stephens, C., Arismendi, T., Wright, M., Hartman, A., Gonzalez, A., Gill, M., et al. (2020). F plasmids are the major carriers of antibiotic resistance genes in human-associated commensal *Escherichia coli*. mSphere, 5, e00310–e00320. doi: 10.1128/mSphere.00310-20
Teng, X., & Hai-Hui, H. (2023). Progress on mechanisms of antibiotic resistance in *Clostridioides difficile*. Yi Chuan = Hereditas, 45(11), 1028–1038. doi:10.16288/j.yczz.23-206.
Ulusoy, B. H., and Chirkena, K. (2019). Two perspectives of *Listeria monocytogenes* hazards in dairy products: The prevalence and the antibiotic resistance. Food Qual. Saf., 3, 233–241. doi: 10.1093/fqsafe/fyz023
Wang, X., Biswas, S., and Ghosh, S. (2019). Biological insights and mechanisms of antibiotic resistance in *Salmonella*: A review of the literature. Microb. Drug Resist., 25, 1042–1054. doi: 10.1089/mdr.2018.0154
Wójcicki, M., Nowaczek, A., and Chajęcka-Wierzchowska, W. (2021). *Salmonella* spp. as a significant challenge to public health: The role of antimicrobial resistance. J. Vet. Res., 65, 271–279. doi: 10.2478/jvetres-2021-0036
