# Supplementary material for: Non-antibiotic therapies for multidrug-resistant gastrointestinal infections: an overview of the use of probiotics, natural compounds, and bacteriophages
Source: Front Antibiot. 2025 May 6;4:1554061. doi: 10.3389/frabi.2025.1554061 (PMC12089134; doi:10.3389/frabi.2025.1554061)
Supplement: Supplementary file 2 [file Table2.docx]

**Supplementary Table 2.** Natural compounds and their actions against MDR-GI pathogens.

| **Category** | **Natural Compound** | **Main Primary Activity** | **Effective Against** | **References** |
| --- | --- | --- | --- | --- |
| Polyphenols | Curcumin  (extracted from *Curcuma longa*) | - Attenuates virulence factors (biofilm production, cell adhesion to epithelial cells and quorum sensing) | - *C. difficile* - *E. coli* - *H. pylori* | Dai et al., 2022  Ray et al., 2021  Adamczak et al., 2020  Mody et al., 2020  Itzia Azucena et al., 2019 |
|  | Tannins  (extracted from tea, berries, and nuts) | - Inhibits enzyme activity - Inhibits fatty acid biosynthesis - Inhibits iron chelation - Disrupts membrane/cell wall structure - Attenuates virulence factors (biofilm and toxins production, cell adhesion to epithelial cells and quorum sensing) | - *C. difficile* - *E. coli* - *H. pylori* - *Salmonella* spp. | Shu et al., 2022  Farha et al., 2020  Puljula et al., 2020 |
|  | Resveratrol  (extracted from grapes and red wine) | - Attenuates virulence factors (biofilm production and quorum sensing) | - *C. difficile* - *E. coli* - *H. pylori* - *Salmonella* spp. | Di Fermo et al., 2020  Vestergaard et al., 2019 |
|  | Catechins  (extracted from tea) | - Inhibit enzymatic activity - Interferes with DNA stability and replication (histone binding) - Attenuates virulence factors (biofilms production and cell adhesion to epithelial cells) | - *E. coli* | Wu et al., 2022  Kim et al., 2021  Raj et al., 2021  Ouyang et al., 2020 |
|  | Gallic acid, Protocatechuic acid, Vanillic acid | - Disrupts membrane/cell wall structure - Inhibits efflux pumps - Attenuates virulence factors (biofilms production and cell adhesion to epithelial cells) | - *E. coli* - *Salmonella* spp. | Tian et al., 2022  Alvarado-Martinez et al., 2020 |
| Flavonoids | Quercetin  (extracted from onions, apples, and berries) | - Inhibits bacterial growth - Attenuates virulence factors (biofilm production, cell adhesion to epithelial cells and quorum sensing) | - *E. coli* - *Salmonella* spp. | Kim et al, 2022  Xue et al., 2019 |
|  | Naringenin  (extracted from *Citrus* fruits) | - Inhibits efflux pumps - Disrupts membrane/cell wall structure - Disrupt fatty acid biosynthesis - Attenuates virulence factors (biofilms production) | - *H. pylori* | Duda-Madej et al., 2020  Tran Trung et al., 2020 |
|  | Apigenin  (extracted from *Petroselinum crispum* and *Matricaria chamomilla*) | - Inhibits bacterial DNA gyrase - Attenuates virulence factors (biofilms production) - Induces reactive nitrogen species, nitric oxide, and superoxide anion production | - *E. coli* - *H. pylori* | Kim et al., 2020  González et al., 2019 |
| Essential Oils | Thymol  (extracted from *Thymus vulgaris*) | - Disrupts membrane/cell wall structure - Interferes with protein regulation - Interferes with DNA synthesis - Attenuates virulence factors (biofilms production) | - *C. difficile* - *E. coli* - *Salmonella* spp. | Zhang et al., 2022  Giovagnoni et al., 2020  Gómez-García et al.,  2020  González et al., 2020 |
|  | Carvacrol  (extracted from *Origanum vulgare*) | - Disrupts membrane/cell wall structure - Attenuates virulence factors (biofilms production) | - *E. coli* - *Salmonella* spp. | Giovagnoni et al., 2020  Trevisan et al., 2020 |
|  | Eugenol  (extracted from *Syzygium aromaticum*) | - Disrupts membrane/cell wall structure - Attenuates virulence factors (biofilms production) | - E. coli - H. pylori - Salmonella spp. | Aljuwayd et al., 2023  Elbestawy et al., 2023  Guimarães et al., 2019 |
|  | Cinnamaldehyde  (extracted from *Cinnamomum verum*) | - Disrupts membrane/cell wall structure - Attenuates virulence factors (biofilms and colibactin production) | - *E. coli* - *Salmonella* spp. | Pereira et al., 2021  Kosari et al., 2020 |
|  | Linalool  (extracted from *Coriandrum sativum* and *Ocimum basilicum*) | - Disrupts membrane/cell wall structure - Attenuates virulence factors (biofilms production) | - *E. coli* - *Salmonella* spp. | Gao et al., 2023  Silva et al., 2021  Jin et al., 2020 |
| Other | Allicin  (extracted from *Allium sativum*) | - Disrupts bacterial membranes - Attenuates virulence factors (biofilms production and quorum sensing) | - E. coli - H. pylori | Borlinghaus et al., 2021  Bhattacharya et al., 2019  Si et al., 2019 |
|  | Berberine  (extracted from *Berberis* species) | - Inhibit DNA replication - Interferes with efflux pumps - Inhibits energy metabolism | - E. coli - H. pylori - Salmonella spp. |  |

**References**

Adamczak, A., Ożarowski, M., & Karpiński, T. M. (2020). Curcumin, a natural antimicrobial agent 531 with strain-specific activity. Pharmaceuticals, 13(7), 153. doi:10.3390/ph13070153
Aljuwayd, M., Almutairi, S. M., Alqahtani, A. M., Alqahtani, M. A., Alqurashi, T. M., Alharbi, M. S., et al. (2023). Biological effects and therapeutic properties of cinnamaldehyde: A review. Molecules 28, 2835. doi: 10.3390/molecules28062835
Alvarado-Martinez, J. R., Salinas-Castro, A., Campos-Rodríguez, R., Rodríguez-Flores, M., and González-Andrade, M. (2020). Flavonoids and their anti-quorum sensing potential: A promising strategy to combat bacterial infections. Molecules 25, 5850. doi: 10.3390/molecules25245850
Bhattacharya, S., Sen, D., and Bhattacharjee, C. (2019). In vitro antibacterial effect analysis of stabilized PEGylated allicin-containing extract from *Allium sativum* in conjugation with other antibiotics. Process Biochem., 87, 221–231. doi: 10.1016/j.procbio.2019.10.011
Borlinghaus, J., Foerster, J., Kappler, U., Antelmann, H., Noll, U., Gruhlke, M. C., et al. (2021). Allicin, the odor of freshly crushed garlic: A review of recent progress in understanding allicin’s effects on cells. Molecules, 26, 1505. doi: 10.3390/molecules26061505
Dai, M., Zhang, C., Feng, M., Wu, Y., and Zhou, Y. (2022). Berberine-based carbon dots enhance intestinal barrier integrity in weaning mice via modulation of the gut microbiota. Front. Nutr. 9, 984870. doi: 10.3389/fnut.2022.984870
Di Fermo, P., De Simone, C., Fracassi, C., Trotta, F., and Cataldi, T. R. I. (2020). The antimicrobial activity of thymol: a natural monoterpene phenol isolated from *Thymus vulgaris* (thyme). Nat. Prod. Res., 34, 1723–1727. doi: 10.1080/14786419.2018.1524292
Duda-Madej, A., Kołodziej, B., Adamski, R., and Kiersnowska, D. (2020). Antibacterial properties of natural flavonoids: Apigenin and luteolin as DNA gyrase inhibitors. J. Mol. Struct., 1220, 128737. doi: 10.1016/j.molstruc.2020.128737
Elbestawy, M. K. M., El-Sherbiny, G. M., and Moghannem, S. A. (2023). Antibacterial, antibiofilm, and anti-inflammatory activities of eugenol clove essential oil against resistant *Helicobacter pylori*. Molecules 28, 2448. doi: 10.3390/molecules28062448
Farha, M. A., French, S., Stokes, J. M., and Brown, E. D. (2020). Bicarbonate alters bacterial susceptibility to antibiotics by targeting the proton motive force. ACS Infect. Dis., 6, 1501–1508. doi: 10.1021/acsinfecdis.0c00008
Gao, M., Li, Q., Lu, J., Guo, M., Chen, Y., and Cui, Z. (2023). Biological activities and mechanisms of allicin in antimicrobial effects: A comprehensive review. Front. Microbiol., 14, 1084367. doi: 10.3389/fmicb.2023.1084367
Giovagnoni, G., Nasuti, C., and Gabbianelli, R. (2020). Eugenol and its anti-inflammatory activity in intestinal epithelial cells. Food Chem. Toxicol., 145, 111688. doi: 10.1016/j.fct.2020.111688
Gómez-García, R. I., López-Hernández, J., Martínez-Ramos, G. L., and Mendoza-García, P. G. (2020). Evaluation of antimicrobial and antibiofilm activities of carvacrol and thymol against foodborne pathogens. Microb. Pathog., 149, 104522. doi: 10.1016/j.micpath.2020.104522
González, J., Herrera-Calderon, O., and Dávalos, A. (2019). Essential oils as inhibitors of bacterial biofilm formation: A promising approach for the control of *Escherichia coli* and *Salmonella* spp. Int. J. Pharm. Sci. Res., 10, 2019–2026. doi: 10.13040/IJPSR.0975-8232.10(5).2019-26
Gonzalez, A., Miñán, A. G., Grillo, C. A., Prieto, E. D., Schilardi, P. L., & de Mele, M. A. F. L. (2020). Characterization and antimicrobial effect of a bioinspired thymol coating formed on titanium surface by one-step immersion treatment. Dental Materials, 36(12), 1495-1507. doi: 10.1016/j.dental.2020.09.006
Guimarães, A. G., Oliveira, M. A., Alves, R. S., Menezes, P. P., Serafini, M. R., Oliveira, R. C. M., et al. (2019). Encapsulation of carvacrol, a monoterpene present in oregano oil, with β-cyclodextrin: effect on the antibacterial activity against *Escherichia coli*. Chem. Biol. Interact., 304, 65–72. doi: 10.1016/j.cbi.2019.03.002
Itzia Azucena, R. C., José Roberto, C. L., Martin, Z. R., Rafael, C. Z., Leonardo, H. H., Gabriela, T. P., et al. (2019). Drug susceptibility testing and synergistic antibacterial activity of curcumin with antibiotics against enterotoxigenic *Escherichia coli*. Antibiotics, 8, 43. doi: 10.3390/antibiotics8020043
Jin, M., Lu, J., Jin, M., and Huang, Q. (2020). The effect of allicin against Helicobacter pylori infection: A systematic review and meta-analysis. Biomed. Pharmacother., 129, 110456. doi: 10.1016/j.biopha.2020.110456
Kim, Y. K., Roy, P. K., Ashrafudoulla, M., Nahar, S., Toushik, S. H., Hossain, M. I. et al (2022). Antibiofilm effects of quercetin against Salmonella enterica biofilm formation and virulence, stress response, and quorum-sensing gene expression. Food Control, 137, 108964. doi: 10.1016/j.foodcont.2022.108964
Kim, J. W., Kim, C. Y., Kim, J. H., Jeong, J. S., Lim, J. O., Ko, J. W., et al. (2021). Prophylactic catechin-rich green tea extract treatment ameliorates pathogenic enterotoxic *Escherichia coli*-induced colitis. Pathogens 10, 1573. doi: 10.3390/pathogens10121573
Kim, S., Woo, E. R., and Lee, D. G. (2020). Apigenin promotes antibacterial activity via regulation of nitric oxide and superoxide anion production. J. Basic Microbiol., 60, 862–872. doi: 10.1002/jobm.202000186
Kosari, M., Samadi, N., and Salehi, R. (2020). Antibacterial properties of linalool against clinical isolates of *Salmonella enterica*. Microb. Pathog., 147, 104383. doi: 10.1016/j.micpath.2020.104383
Mody, D., Athamneh, I. M. A., and Seleem, M. N. (2020). Curcumin: A natural derivative with antibacterial activity against *Clostridium difficile*. J. Glob. Antimicrob. Resist., 21, 154–161. doi: 10.1016/j.jgar.2019.11.008
Ouyang, J., Zhu, K., Liu, Z., and Huang, J. (2020). Prooxidant effects of epigallocatechin-3-gallate in health benefits and potential adverse effects. Oxid. Med. Cell. Longev., 2020, 9723686. doi: 10.1155/2020/9723686
Pereira, W. A., Pereira, C. D. S., Assunção, R. G., da Silva, I. S. C., Rego, F. S., Alves, L. S. R., et al. (2021). New insights into the antimicrobial action of cinnamaldehyde towards *Escherichia coli* and its effects on intestinal colonization of mice. Biomolecules, 11, 302. doi: 10.3390/biom11020302
Puljula, E., Walton, S. P., Manner, S., Aho, J., Huuskonen, J., and Yli-Kauhaluoma, J. (2020). Aminoacridine derivatives as antibacterial agents *against Staphylococcus aureus*. Eur. J. Med. Chem., 186, 111857. doi: 10.1016/j.ejmech.2019.111857
Raj, R., Agarwal, N., Raghavan, S., Chakraborti, T., Poluri, K. M., Pande, G., et al. (2021). Epigallocatechin gallate with potent anti-*Helicobacter pylori* activity binds efficiently to its histone-like DNA binding protein. ACS Omega, 6, 3548–3570. doi: 10.1021/acsomega.0c05230
Ray, A. K., Luis, P. B., Mishra, S. K., Barry, D. P., Asim, M., Pandey, A., et al. (2021). Curcumin oxidation is required for inhibition of *Helicobacter pylori* growth, translocation, and phosphorylation of CagA. Front. Cell. Infect. Microbiol, 11, 765842. doi:10.3389/fcimb.2021.765842.
Shu, J., Liu, H., Liu, Y., Chen, X., Yu, Y., Lv, Q., et al. (2022). Tannic acid inhibits *Salmonella enterica* serovar *Typhimurium* infection by targeting the type III secretion system. Front. Microbiol., 12, 784926. doi:10.3389/fmicb.2021.784926.
Si, H., Zhang, L., Yang, X., and Gong, X. (2019). Anti-bacterial activity and mechanism of protocatechuic acid against Listeria monocytogenes. Food Control, 96, 274–280. doi: 10.1016/j.foodcont.2018.09.026
Silva, C. G., Yudice, E. D. C., Campini, P. A. L., & Rosa, D. S. (2021). The performance evaluation of eugenol and linalool microencapsulated by PLA on their activities against pathogenic bacteria. 99Materials Today Chemistry, 21, 100493. doi: 10.1016/j.mtchem.2021.100493.
Tian, J., Wang, S., Hu, S., Wang, R., and He, Y. (2022). Natural phenolic compounds as efflux pump inhibitors to reduce antimicrobial resistance in bacteria: A review. Crit. Rev. Food Sci. Nutr., 62, 2072–2084. doi: 10.1080/10408398.2020.1841160
Tran Trung, C., Nguyen Thi Thanh, H., Nguyen, T. T. M., and Dang, A. D. (2020). Apigenin as a potential antibiotic against *Helicobacter pylori*: *In vitro* and *in silico* studies. J. Appl. Microbiol., 129, 1035–1045. doi: 10.1111/jam.14644
Trevisan, M., Frazzini, V., Riva, C., Esposito, G., and Uccelli, A. (2020). Eugenol modulates intestinal barrier function and reduces inflammation in a murine model of colitis. Phytother. Res., 34, 2570–2580. doi: 10.1002/ptr.6704
Vestergaard, M., and Ingmer, H. (2019). Antibacterial and anti-inflammatory properties of resveratrol. Int. J. Mol. Sci., 20, 2781. doi: 10.3390/ijms20112781
Wang, X., Biswas, S., and Ghosh, S. (2019). Biological insights and mechanisms of antibiotic resistance in Salmonella: A review of the literature. Microb. Drug Resist., 25, 1042–1054. doi: 10.1089/mdr.2018.0154
Wu, Z., Shen, J., Xu, Q., Xiang, Q., Chen, Y., Lv, L., et al. (2022). Epigallocatechin-3-gallate improves intestinal gut microbiota homeostasis and ameliorates Clostridioides difficile infection. Nutrients, 14(18), 3756. doi:10.3390/nu14183756.
Xue, J., Xie, M., Ma, Y., and Zhang, W. (2019). Mechanism of action of naringenin against *Helicobacter pylori*. Molecules 24, 3222. doi: 10.3390/molecules24183222
Zhang, L., Peng, D., Wang, X., and Tang, Y. (2022). Mechanism of action of carvacrol against *Salmonella enterica*. Food Chem. 367, 130671. doi: 10.1016/j.foodchem.2021.130671
Zhou, H., Wang, W., Cai, L., and Yang, T. (2023). Potentiation and mechanism of berberine as an antibiotic adjuvant against multidrug-resistant bacteria. Infect. Drug Resist. 16, 7313–7326. doi: 10.2147/IDR.S426805
Zhou, T., Wu, Y., Sun, Y., Meng, Y., Xu, J., Xu, J., et al. (2022). Synergistic effect of curcumin with antibiotics against multidrug-resistant *Salmonella enterica* serovar *Typhimurium*. Front. Microbiol. 13, 884168. doi: 10.3389/fmicb.2022.884168
